# Population structure and evolutionary history of the greater cane rat (*Thryonomys swinderianus*) from the Guinean Forests of West Africa

**DOI:** 10.3389/fgene.2023.1041103

**Published:** 2023-02-27

**Authors:** Isaac A. Babarinde, Adeniyi C. Adeola, Chabi A. M. S. Djagoun, Lotanna M. Nneji, Agboola O. Okeyoyin, George Niba, Ndifor K. Wanzie, Ojo C. Oladipo, Ayotunde O. Adebambo, Semiu F. Bello, Said I. Ng’ang’a, Wasiu A. Olaniyi, Victor M. O. Okoro, Babatunde E. Adedeji, Omotoso Olatunde, Adeola O. Ayoola, Moise M. Matouke, Yun-yu Wang, Oscar J. Sanke, Saidu O. Oseni, Christopher D. Nwani, Robert W. Murphy

**Affiliations:** ^1^ Shenzhen Key Laboratory of Gene Regulation and Systems Biology, School of Life Sciences, Southern University of Science and Technology, Shenzhen, China; ^2^ Department of Biology, School of Life Sciences, Southern University of Science and Technology, Shenzhen, China; ^3^ State Key Laboratory of Genetic Resources and Evolution, Kunming Institute of Zoology, Chinese Academy of Sciences, Kunming, China; ^4^ Sino-Africa Joint Research Centre, Chinese Academy of Sciences, Kunming, China; ^5^ Centre for Biotechnology Research, Bayero University, Kano, Nigeria; ^6^ Laboratory of Applied Ecology, Faculty of Agronomic Sciences, University of Abomey-Calavi, Cotonou, Benin; ^7^ Department of Ecology and Evolutionary Biology, Princeton University, Princeton, NJ, United States; ^8^ National Park Service Headquarters, Federal Capital Territory, Abuja, Nigeria; ^9^ National Centre for Animal Husbandry and Veterinary Training, Jakiri, North West Region, Cameroon; ^10^ Department of Zoology, University of Douala, Douala, Cameroon; ^11^ Department of Zoology, Faculty of Life Sciences, University of Ilorin, Ilorin, Kwara State, Nigeria; ^12^ Old Oyo National Park, Oyo, Nigeria; ^13^ Animal Genetics & Biotechnology, Federal University of Agriculture, Abeokuta, Nigeria; ^14^ Department of Animal Genetics, Breeding and Reproduction, College of Animal Science, South China Agricultural University, Guangzhou, China; ^15^ Department of Animal Science, Faculty of Agriculture, Adekunle Ajasin University, Akungba-Akoko, Ondo State, Nigeria; ^16^ Department of Animal Science and Technology, School of Agriculture and Agricultural Technology, Federal University of Technology, Owerri, Nigeria; ^17^ Department of Zoology, University of Ibadan, Ibadan, Oyo State, Nigeria; ^18^ Department of Fisheries and Aquatic Resources Management, University of Buea, Buea, Cameroon; ^19^ Wild Forensic Center, Kunming, China; ^20^ Taraba State Ministry of Agriculture and Natural Resources, Jalingo, Nigeria; ^21^ Department of Animal Sciences, Faculty of Agriculture, Obafemi Awolowo University, Ile-Ife, Nigeria; ^22^ Department of Zoology and Environmental Biology, Faculty of Biological Sciences, University of Nigeria, Nsukka, Nigeria; ^23^ Centre for Biodiversity and Conservation Biology, Royal Ontario Museum, Toronto, ON, Canada

**Keywords:** genetic diversity, population structure, lower guinean forests, mitochondrial sequences, *Thryonomys swinderianus*

## Abstract

Grasscutter (*Thryonomys swinderianus*) is a large-body old world rodent found in sub-Saharan Africa. The body size and the unique taste of the meat of this major crop pest have made it a target of intense hunting and a potential consideration as a micro-livestock. However, there is insufficient knowledge on the genetic diversity of its populations across African Guinean forests. Herein, we investigated the genetic diversity, population structures and evolutionary history of seven Nigerian wild grasscutter populations together with individuals from Cameroon, Republic of Benin, and Ghana, using five mitochondrial fragments, including D-loop and cytochrome b (*CYTB*). D-loop haplotype diversity ranged from 0.571 (± 0.149) in Republic of Benin to 0.921 (± 0.013) in Ghana. Within Nigeria, the haplotype diversity ranged from 0.659 (± 0.059) in Cross River to 0.837 (± 0.075) in Ondo subpopulation. The fixation index (F_ST_), haplotype frequency distribution and analysis of molecular variance revealed varying levels of population structures across populations. No significant signature of population contraction was detected in the grasscutter populations. Evolutionary analyses of *CYTB* suggests that South African population might have diverged from other populations about 6.1 (2.6–10.18, 95% CI) MYA. Taken together, this study reveals the population status and evolutionary history of grasscutter populations in the region.

## 1 Introduction

Grasscutter or greater cane rat (*Thryonomys swinderianus*) is one of the two known extant cane rats found exclusively in sub-Saharan Africa (López-Antoñanzas et al., 2004; Woods and Kilpatrick, 2005; Hoffmann, 2008). Indeed, grasscutter and the lesser cane rat (*T. gregorianus*) are the only known extant members of the genus *Thryonomys* and the family Thryonomidae (Woods and Kilpatrick, 2005; Merwe, 2015). Thrynomidae, Petromuridae, and Bathyergidae make up Phiomorpha, one of the early colonization of Hystricognaths and even rodents in African (Huchon and Douzery, 2001; Poux et al., 2006). Fossil evidence suggests that Phiomorpha might have had many members that are now extinct (Bate, 1947; López-Antoñanzas et al., 2004; Kraatz et al., 2013; Sallam and Seiffert, 2016; Sallam and Seiffert, 2020). However, the cane rats, the dassie rats, and the blesmols are probably the only known extant species of Phiomorpha (Huchon and Douzery, 2001; Sallam et al., 2009; Sallam and Seiffert, 2016), suggesting that these species must have evolved strong adaptive traits. Despite the relatively few species in Phiomorpha, the phylogeny is still under debate (D’Elía et al., 2019; Sheng et al., 2020). Among the known extant Phiomorpha species, the cane rats, especially the grasscutter (also called the greater cane rat), has the largest body size (Baptist and Mensah, 1986a). Despite the large body size, grasscutter is a good runner and swimmer; hence, it has a relatively wider geographical distribution (Woods and Kilpatrick, 2005; Hoffmann, 2008) than several other Phiomorpha species. Consequently, cane rats, mainly the grasscutters have been hunted for their meat across many countries in sub-Saharan Africa (Baptist and Mensah, 1986a; Kalu and Aiyeloja, 2002; Annor et al., 2008; Andem, 2012; Coker et al., 2017; Yisau et al., 2019).

One significant physical difference between the grasscutter and the lesser cane rats is their body size. Grasscutter can weigh up to 6 kg (Baptist and Mensah, 1986a; Baptist and Mensah, 1986b; Van Der Merwe, 1999; Merwe, 2015), but the lesser cane rat weighs less. The bigger body weight, unique meat flavor and the relative abundance of grasscutter in West Africa have made the animal an important source of animal proteins for human populations, especially in the rural areas. Grasscutter is an important game animal with desirable meat qualities (Kalu and Aiyeloja, 2002; Yisau et al., 2019; Teye et al., 2020), therefore it has attracted considerable scientific interests (Annor et al., 2008; Owusu et al., 2010; Andem, 2012; Adu et al., 2017; Coker et al., 2017; Yisau et al., 2019; Durowaye et al., 2021). Efforts are now being made to improve its domestication as micro-livestock, while the wild populations are continuously being hunted for human consumption.

Grasscutters are naturally adapted to the reeds and sugar cane farms, but recent human anthropogenic activities have drastically made them adapt to a wide range of habitats, including even urban areas (Wilson and Reeder, 2005; Hoffmann, 2008; Kilwanila et al., 2021). However, their distribution has been somewhat limited to certain parts of sub-Saharan Africa (López-Antoñanzas et al., 2004; Hoffmann, 2008; Coker et al., 2017). They are common animal pests found in grasslands and cultivated forest regions of sub-Saharan Africa (Fayenuwo and Akande, 2002), posing a threat of huge economic loss to the crop farmers. Consequently, in addition to the meat, another motivation for grasscutter hunting is for pest control (Fayenuwo and Akande, 2002; Aluko et al., 2015; Essuman and Duah, 2020) (Fayenuwo and Akande, 2002; Aluko et al., 2015; Essuman and Duah, 2020). Therefore, the animals have a great economic importance both in agriculture and human dietary animal protein supply chain (Adenyo et al., 2012).

Generally, animals with large body sizes tend to have smaller litter size and fewer litter frequency (Tuomi, 1980; Babarinde and Saitou, 2020). The average litter size of grasscutter is 2.9 (Van Der Merwe, 1999; Adu et al., 2000), while the maximum of two litters per female is reported per year (Van Der Merwe, 1999)^.^ The relatively low reproductive rates of this animal and high hunting intensity with no regulations (Van Der Merwe, 1999; Andem, 2012; Yisau et al., 2019; Teye et al., 2020) should suggest grasscutter to be among the threatened or endangered wildlife species (Aluko et al., 2015). However, grasscutter is classified as “Least Concern” animal by the International Union for Conservation of Nature (Child, 2016), implying that the animal does not require any urgent conservation efforts (Hoffmann, 2008; Adenyo et al., 2012).

Previous studies on grasscutter at the population genetics level are scarce and with limited scope. For example, Adenyo et al. (2013) studied exclusively Ghanaian grasscutter population using mitochondrial D-loop region, while other studies have employed microsatellite markers (Adenyo et al., 2017; Coker et al., 2017). Another study on bush meat included grasscutter mitochondrial markers (Gaubert et al., 2015) but did not focus on grasscutter population genetics. It is noteworthy that study on grasscutter population genetics using mitochondrial nucleotide sequences in Nigeria is yet to be documented (Mustapha et al., 2020). Importantly, the impacts and the threat of hunting to the wild grasscutter population has not been extensively investigated. Focusing on Nigeria, the country with the largest land area in West Africa, the largest human population in Africa and potentially higher threat on wild grasscutter population due to hunting, this study aimed at investigating the wild grasscutter populations in and around Nigeria. The analyses of the demographic histories would reveal any potential threat to the wild grasscutter populations. Also, maximizing breeding gains from grasscutter requires adequate understanding of the genetic diversity and the population structures of the wild populations from where the breeding stock would be selected. Therefore, we analyzed the mitochondrial genome sequences to investigate the population structures and history of wild grasscutter populations in Nigeria and neighboring countries in the African Guinea forests including Republic of Benin, Cameroon, and Ghana. Our results not only help understand the genetic diversity and population structures of Nigerian wild grasscutter populations, but also provide insights into potential evolutionary history of wild grasscutter populations in the African Guinea forests. The findings from the study would provide valuable insights into wild grasscutter breeding stock selection for the purpose of domestication.

## 2 Materials and methods

### 2.1 Ethical statement

*T. swinderianus* is not protected under any legislation and not considered threatened or endangered. Samples from Nigeria were collected through capture and release from National Parks and permissions were collected from the Nigerian National Park Service (NPH/GEN/121/XXV/675). Samples from Republic of Benin (586/DGEFC/DCPRN/SCPRN/SA) and Cameroon were collected from bush meat markets. We have complied with ARRIVE at submission.

### 2.2 Sample collection

**FIGURE 1:**
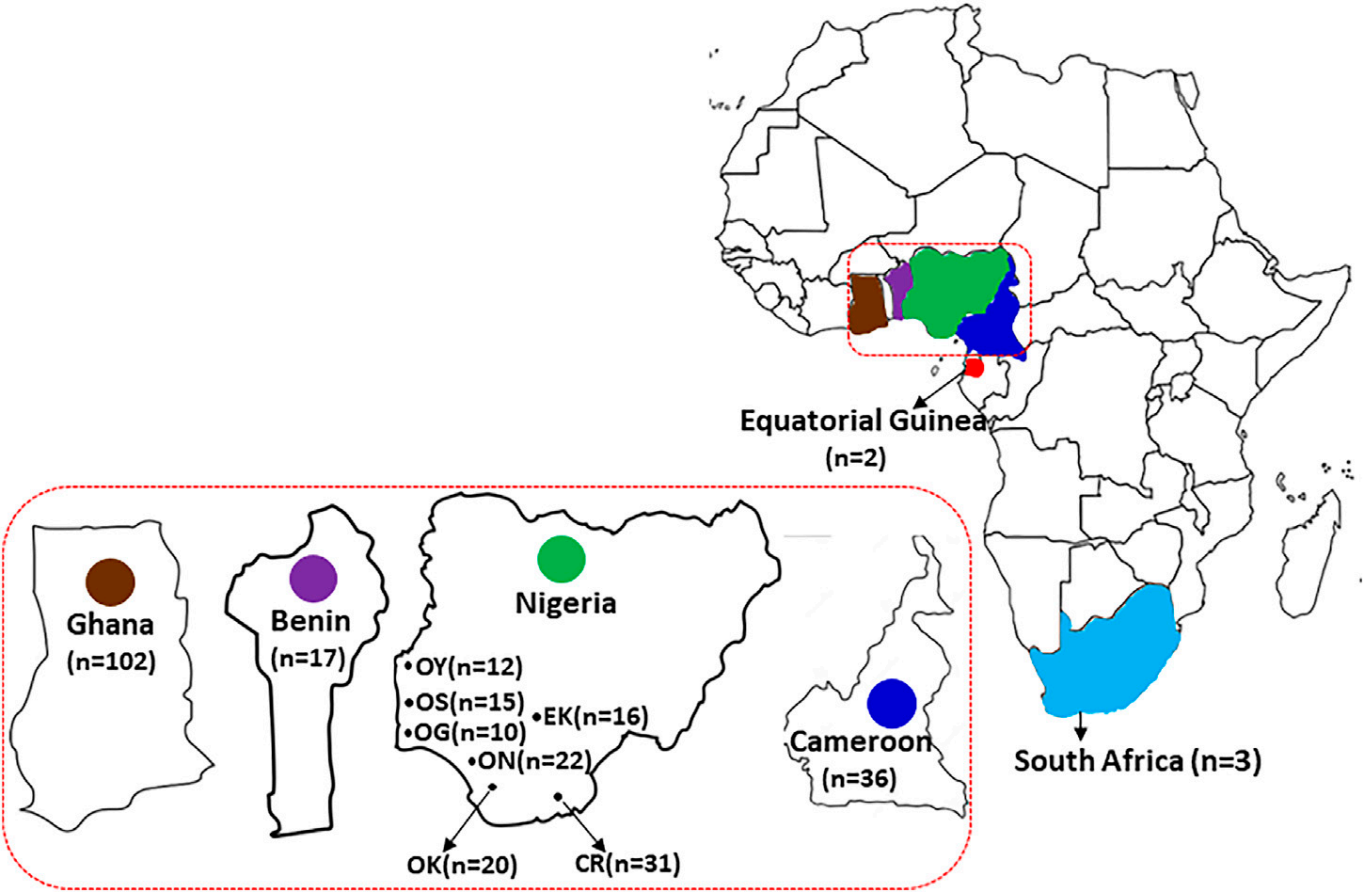
The geographical distribution of the grasscutter populations. The samples were collected from countries in the Guinean Forests of West Africa, including Republic of Benin, Nigeria, and Cameroon. Nigerian samples were collected from seven different states. Sequences of samples collected from Ghana, Equatorial Guinea and South Africa were also analyzed.

Hair samples plucked to the root were collected from 121 wild grasscutters sampled from seven Nigerian states which belong to three different vegetational zones that support wild grasscutter populations. The sampling locations which are areas with intense grasscutter hunting included Oyo and Ekiti (derived savanna), Osun, Ogun, Ondo and Edo (rain forest) and Cross River (humid rainforest) states (Figure 1). Five of the locations are in the Southwest zone while Edo and Cross River states are in the South-South zones of Nigeria. Additional individuals were sampled from bush meat markets across different vegetational zones in Cameroon (*n* = 36) and Republic of Benin (*n* = 17). All samples were collected from January to March 2019 (Supplementary Tables S1). Hair root samples collected were preserved in 95% ethanol and stored under −80°C at the State Key Laboratory of Genetic Resources and Evolution, Kunming Institute of Zoology, Chinese Academy of Sciences, China.

### 2.3 DNA extraction, PCR amplification and sequencing

Genomic DNA extractions were performed following the standard phenol-chloroform method (Sambrook and Russell, 2001; Akinwole and Babarinde, 2019). The extracted DNA was quantified using the Thermo Scientific^™^ NanoDrop 2000 spectrophotometer to assess purity. Furthermore, the DNA extracts were checked for molecular quality by running them through a 2% agarose gel together with a 2 kb DNA ladder marker. The five mitochondrial fragments were sequenced in 175 grasscutters samples using primer pairs amplifying 384–658 bp fragments of D-loop, cytochrome b (*CYTB*), cytochrome c oxidase I (*COI*), ribosomal subunits 12 S and 16 S (Supplementary Tables S1, S2). The amplification was performed on a GeneAmp^®^ PCR system 9,700 Applied Biosystems in a 50 µL volume containing PCR mixture of 5 µL 10x reaction buffer, 1.5 mM MgCl2, 0.2 mM dNTPs, 0.2 µM each primer, 1.5 µ Taq DNA polymerase (TaKaRa), and approximately 30 ng genomic DNA. PCR cycling conditions included an initial denaturation of 95°C for 2 min, followed by 35 cycles of 95°C for 30 s, annealing (30 s; see Supplemetary Table S2 for T), an extension of 72°C for 30 s and a final extension of 72°C for 15 min. The PCR products were purified with ExoSAP-IT as per the manufacturer’s instructions (Affymetrix). Sequencing reactions were performed using the BigDyeTM Terminator Cycle Sequence Kit 3.1 Ready Reaction Cycle Sequencing Kit (ABI Applied Biosystems), and the products were purified by alcohol precipitation. The purified products were analysed in ABI PRISM 3730 automated DNA sequencer (ABI Applied Biosystems). The electropherograms for each sequence were visualized, edited, and aligned by SeqMan Pro of DNASTAR Lasergen 7.1.0 (DNAStar Inc., Madison, WI) with the reference sequence Accession AJ301644 (Mouchaty et al., 2001).

### 2.4 Dataset assembly

The identities of the newly generated sequences were confirmed by BLAST searches (Altschul et al., 1997) in the National Center for Biotechnology Information (https://blast.ncbi.nlm.nih.gov/Blast.cgi). The nucleotide sequences of all the newly sequenced samples were deposited in GENBANK under accession numbers MZ418538 - MZ418687; MZ418390 - MZ418537; MZ418252 - MZ418389; MZ418839 - MZ418996; MZ418688 - MZ418838 for D-loop, *CYTB*, *COI*, ribosomal subunits 12 S and 16 S respectively. In addition, previously published D-loop (*n* = 86), *CYTB* (*n* = 25), *COI* (*n* = 26), 12 S ribosomal subunit (*n* = 22) and 16 S (*n* = 27) sequences of *T. swinderianus* from Ghana, Nigeria, Equatorial Guinea, and South Africa (Mouchaty et al., 2001; Gaubert et al., 2015) were downloaded from the NCBI database (http://www.ncbi.nlm.nih.gov) (Supplementary Table S1). Further, the nucleotide and amino acid sequences of multiple rodent species were downloaded from the NCBI database (http://www.ncbi.nlm.nih.gov). In total, 236 D-loop, 173 *CYTB*, 164 *COI*, 180 ribosomal subunits 12 S and 178 16 S nucleotide sequences of *T. swinderianus* were analysed in this study. The *CYTB* sequences of dassie rat (*Petromus typicus*, accession number DQ139935.1), naked mole rat (*Heterocephalus glaber*, accession number NC_015112.1) and guinea pig (*Cavia porcellus*, accession number NC_000884.1) were also included in the estimation of divergence times.

### 2.5 Initial data analyses and sequence alignment

First, the sequences of each region were aligned in MEGA7 (Kumar et al., 2016) using CLUSTALX 2.1 (Larkin et al., 2007) with default parameters. For quality assessment, the aligned sequences of *CYTB* and *COI* were independently translated into amino acids using the vertebrate mitochondrial code. No premature stop codons were observed, demonstrating that the open reading frame was maintained in the protein-coding loci. In all the loci, no unexpected gap was found within the alignments.

### 2.6 Population analyses

#### 2.6.1 Genetic diversity

Sequence comparison and identification of haplotypes were performed with DNASP 5.10.1 (Librado and Rozas, 2009). Genetic diversity was estimated using Arlequin v3.5 (Excoffier and Lischer, 2010) and expressed in terms of number of haplotypes (nHT), haplotype diversity (HTdiv), nucleotide diversity (πdiv), mean number of pairwise differences (MNPD) and their respective standard deviations estimated across all populations used in this study. We ran the analyses on the five concatenated regions for Nigerian samples. Based on the result of the PartitionFinder2, we further analysed the two clusters of mitochondrial regions for Nigeria and cross-countries samples. Because all the five regions were not concurrently sequenced in the same Ghana samples (Supplementary Table S1), all the five mitochondrial regions could not be combined for cross-country analyses.

#### 2.6.2 Phylogenetic analyses

Because of the limited number of variable sites, we explored the possibility of merging all the five regions. We first evaluated the molecular evolution models for the five mitochondrial regions using PartitionFinder2 (Lanfear et al., 2012). The best partition scheme, ranked by the Bayesian information criterion (BIC), separated the regions into two clusters. The first cluster included D-loop while the other four regions were clustered together. The best evolutionary model for the two clusters was HKY (Hasegawa et al., 2005) with invariant site (HKY + I). We therefore analysed the D-loop independently. The other four regions (*CYTB*, *COI*, 12 S and 16 S), which constituted the second partition, were concatenated and further analysed as a unit.

The phylogenetic analyses were conducted using both maximum likelihood (Felsenstein, 1981) and Bayesian inference (Rannala and Yang, 1996; Mau and Newton, 1997) methods. First, the best evolutionary model for each multiple sequence alignment was determined using PartitionFinder2 or MEGA7 (Kumar et al., 2016). The phylogenetic trees were then constructed with the selected best-fit evolutionary models. The maximum likelihood phylogenetic analyses were conducted in MEGA7 with bootstrap test set at 1,000 replications to assess the confidence of the nodes (Felsenstein, 1985). The Bayesian inference trees were constructed with BEAST v2.6.6 (Bouckaert et al., 2019). The priors were set using BICEPS model (Douglas et al., 2021; Bouckaert, 2022). Strict clock with clock rate of 1.0 was used. The MCMC chain length of 50 million was used. Pre-burnin was set to 10% of the total run, while the number of initialization attempts was set to 1,000, with every 1,000 samples being stored. The appropriateness of the MCMC run was evaluated with Tracer v1.7.2 (Rambaut et al., 2018). TreeAnnotator in BEAST package was used to analyze the trees to obtain the tree with the maximum clade credibility based on median heights. To further visualize the genetic relationships between the haplotypes, we constructed a median-joining network (MJ) (Bandelt et al., 1999) using the default setting of weights of both transversions and transitions as implemented in NETWORK 4.6.11 software (http://www.fluxus-engineering.com). When needed, the networks were cleaned using maximum parsimony (MP) options.

#### 2.6.3 Demographic dynamic profiles and population genetic structure parameters

To investigate the demographic patterns and population dynamics of the grasscutter populations, demographic statistical parameters for Tajima’s *D* (Tajima, 1989), Fu’s *Fs* (Fu, 1997) and Harpending raggedness (Harpending, 1994), and population F_ST_ were calculated using ARLEQUIN v3.5.1.3 (Excoffier and Lischer, 2010). To further investigate the signatures of population structure, the haplotype frequencies of the populations were compared (Raymond and Rousset, 1995). Additionally, population haplotype mismatch distribution patterns were estimated (Rogers and Harpending, 1992). To further infer the genetic variation within populations, among populations, and groups of the grasscutter populations, analysis of molecular variance (AMOVA) was conducted with 50,000 permutations in ARLEQUIN v3.5 software. This analysis was conducted at various hierarchical levels. The significant levels for each hierarchical cluster tested were evaluated using the F_ST_ parameter at a significant *p* level of 0.05.

For the Bayesian inference of population size dynamics, the control file was generated with Beauti and the analysis was carried out in BEAST2 (Bouckaert et al., 2019). Based on the result of PartitionFinder2, HKY evolutionary model (Hasegawa et al., 2005) was used. There was no sufficient fossil and nucleotide sequence data to estimate the substitution rate. We also could not use substitution rate of other rodent species because the analyses of nuclear DNA sequences have revealed heterogeneity in rodent evolutionary rates (Babarinde and Saitou, 2013, 2020). Consequently, we used strict clock with clock rate of 1.0, and left the results in substitution rate units. The substitution rate, proportion of invariant sites, Kappa and frequencies were estimated from the data. The priors were set using default values of the BICEPS model (Douglas et al., 2021; Bouckaert, 2022). The MCMC chain lengths of 10, 50, 75 and 100 million were used, depending on the data. Pre-burnin was set to 10% of the total run, while the number of initialization attempts was set to 1,000, with every 1,000 samples being stored. The appropriateness of the MCMC run was evaluated with Tracer v1.7.2 (Rambaut et al., 2018), and the MCMC chain length was increased if there was need. The trace and the tree files were analysed using Bayesian Skyline Analyses in Tracer. Defaults values were used, except for the maximum time being set to “median”.

#### 2.6.4 Relationship between the genetic distance and the geographical distance

Because some of the samples were collected from meat markets, it was difficult to obtain the exact locations of all the samples. Hence, only the approximate locations were used, assuming that the animals sampled at a market were hunted from a location close to the market. We followed the procedure used in a Muscovy duck study (Adeola et al., 2022). For each state or country, we obtained the longitude and latitude of the central location. The coordinates of each pair of the location are then used to infer the geographical distance in kilometers. It is important to stress that this gross approximation of geographical distances would be less accurate in populations that are geographically close. However, we used this approximation as a proxy for the relationship. The relationships between the geographical distance and the genetic distance (F_ST_ values) computed from ARLEQUIN, were then investigated and presented in terms of correlation coefficients and scatter plots. This analysis was done separately for both the four concatenated mitochondrial regions, and the D-loop region.

#### 2.6.5 Genetic component analyses

The analyses of the genetic ancestry were first computed in STRUCTURE version 2.3.4 (Pritchard et al., 2000). We ran the analyses separately for the two partitions. The data were coded such that nucleotides A, C, G and T were coded as “11”, “22”, “33”, and “44”, respectively. Because the mitochondrial genome is not diploid, we coded the second allele as “0” and indicated “0” as the missing data in STRUCTURE. Only the polymorphic positions were used. The analyses were run with the Pre-burnin set to 5,000 before 10,000 MCMC replications. Models with or without admixture were tested, with alpha value inferred from the data, starting from 1.0. Independent allele frequency was selected with lambda set to 1. The analyses were run in 10 iterations for k = 2 to 11 for all populations. In addition, model involving migration was also tested. To test various values of k, we first checked the estimated log probability of data. We then focused on the k value with the highest Δk (Evanno et al., 2005). We ran the analysis separately for the four merged regions (*COI*, *CYTB*, 12 S and 16 S) and D-loop.

We further investigated the genetic components with the discriminant analysis of principal components (DAPC) (Jombart et al., 2010) implemented in adegenet package (Jombart, 2008). Focusing on the polymorphic positions, the data were coded such that A, C, G and T were represented as 1, 2, 3 and 4, respectively. The matrix of the positions, with individuals as rows and positions as columns, was then made. The matrix was converted to the DAPC data using *df2genind* in adegenet package. The data were first converted into principal components (PCs). The top 30 *p*Cs were used for the analysis. The PC-transformed data were then used as inputs for DAPC. The results were presented using *compoplot* in adegenet package.

#### 2.6.6 Population phylogenetic tree

The population phylogenetic trees were made for the four concatenated and D-loop regions using POPTREE2 (Takezaki et al., 2010). Corrected F_ST_ values were used for the computation of distances while NJ method (Saitou and Nei, 1987) was used for the phylogenetic reconstruction. Bootstrap test with 1,000 replications was perform to test the reliability of the branches.

#### 2.6.7 Pairwise nucleotide distances and principal component analysis

The pairwise nucleotide distances were computed with MEGA7 using maximum composite likelihood method with 5 Gamma parameters. Bootstrap method with 1,000 replicates was used for the estimation of variance. The principal component analysis (PCA) was computed according to the procedure reported by Babarinde and Saitou (2020). Briefly, pairwise genetic distances estimated from MEGA7 were converted into a full matrix. The *prcomp* in R base (https://www.R-project.org/) was then used to compute the PCA from the pairwise distances.

### 2.7 Divergence time estimates

Mitochondrial *CYTB* haplotypes of grasscutter samples were used for the estimation of the divergence times. In addition, the *CYTB* sequences of dassie rats (*Petromus typicus*, accession number DQ139935.1), naked mole rat (H*eterocephalus glaber*, accession number NC_015112.1) and guinea pig (*Cavia porcellus*, accession number NC_000884.1) were also retrieved. The best evolutionary model for the aligned sequences was checked. The divergence time estimates were then computed using *BEAST (Heled and Drummond, 2010) implemented in the v2.6.6 of BEAST (Bouckaert et al., 2019). Multispecies coalescent model was used for the estimation (Heled and Drummond, 2010; Barido-Sottani et al., 2018; Zhang et al., 2018). For site model, TN93 (Tamura and Nei, 1993) was used with Gamma Category Count of 5, Shape estimated from the data, starting with 1. Kappa1 and Kappa2 were both estimated starting from 2.0. Empirical frequencies were used. Random local clock model with scaling was used. The clock rate was set to be estimated from the data. For the multispecies coalescent model, the population mean of the species population size was estimated from the data starting from 1.0, with linear population function. The ploidy for Y or mitochondrial was used.

The priors for the multispecies coalescent models were set as follows. Yule model was used for the initial tree. Poisson distribution was assumed for the rate changes. For all the other parameters, log Normal distributions were assumed with the values estimated from the data. The calibration points used include 17.6–28.1 million year divergence [M = 3.14, S = 0.12] for dassie rats and cane rats (Fabre et al., 2012; Patterson and Upham, 2014; Upham and Patterson, 2015), 32.6–39.4 million-year divergence [M = 3.59, S = 0.05] for naked mole rats and cane rats (Patterson and Upham, 2014; Upham and Patterson, 2015) and 41.4–49.5 million-year divergence [M = 3.82, S = 0.04] for guinea pigs and cane rats (Poux et al., 2006; Phillips, 2015; Upham and Patterson, 2015). All the calibration branches were treated as monophyletic with log Normal distributions. The chain length of the MCMC run was 100 million with the tree sampled at every 1,000 runs. Pre-burnin was 5 million. The MCMC analysis was evaluated with the Tracer. The consensus tree was then made by TreeAnnotator in BEAST package. The tree with the maximum clade credibility based on median heights was selected using burnin percentage of 10%. The tree was visualized using FigTree (tree.bio.ed.ac.uk/software/figtree).

## 3 Results

### 3.1 Genetic structure of Nigerian grasscutter populations with concatenated mitochondrial regions

**FIGURE 2:**
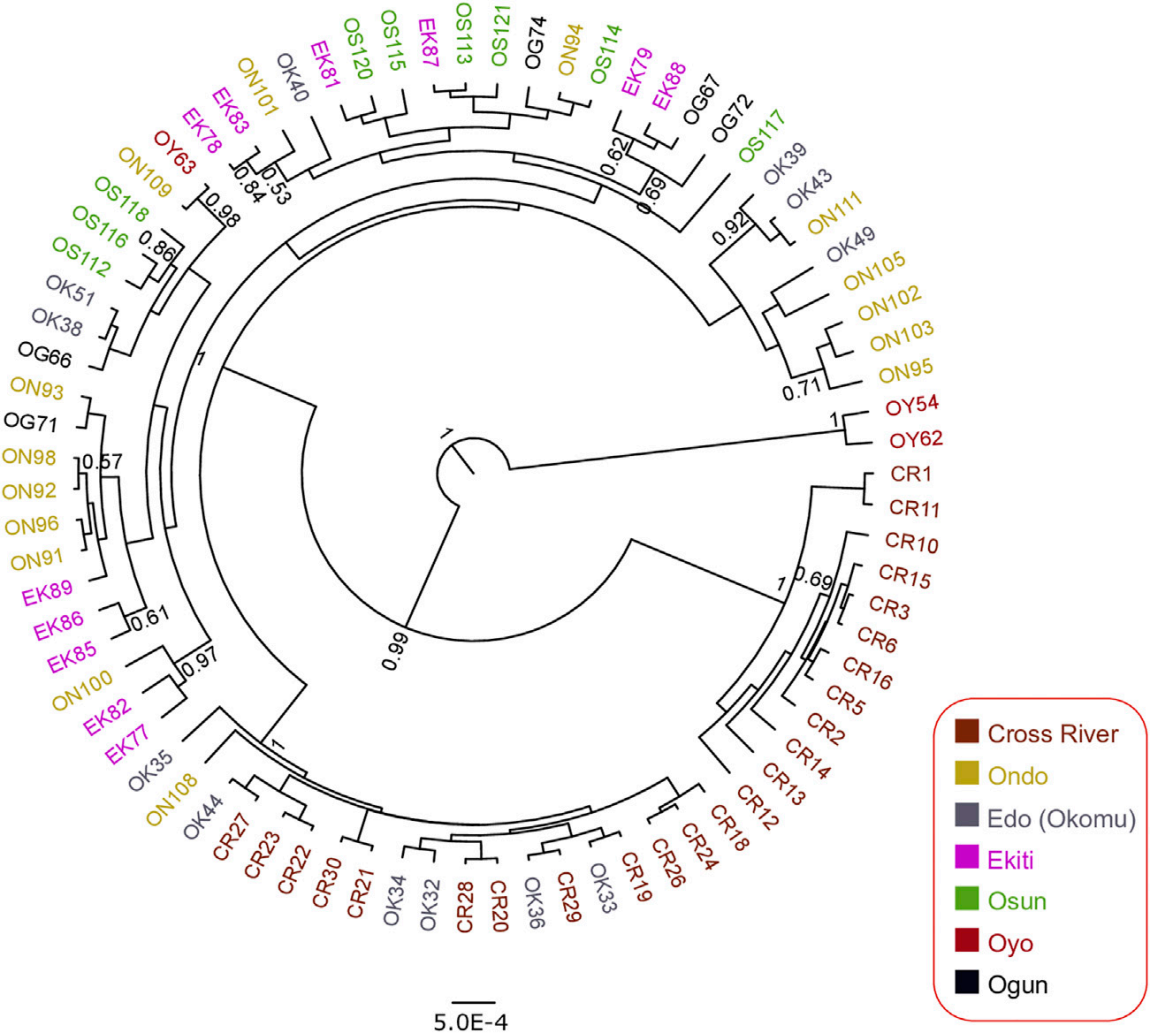
Bayesian phylogenetic tree for 79 Nigerian wild grasscutter samples based on the 2,437 bp of the five concatenated mitochondrial regions. The samples are colored by the sampling location. The rooted tree with the maximum clade support from 45,000 MCMC trees is presented. Branches with at least 0.5 posteriors are shown.

To investigate the genetic status of the Nigerian wild grasscutter populations, we first investigated the phylogenetic relationships among the individuals sampled across the investigated locations (Figure 1, Supplementary Tables S1). To maximize the number of informative sites, we assessed the evolutionary models of the five mitochondrial regions. The results of the PartititionFinder2 showed that the five regions could be clustered into two schemes, both having HKY + I as the best model (Supplementary Figure S1A). Although the pairwise genetic distances varied across regions (Supplementary Figure S1B), we decided to concatenate the five mitochondrial regions for the Nigerian populations because all the schemes had the same evolutionary model (Supplementary Figure S1A). The phylogenetic trees made by Bayesian inference (Figure 2) and maximum likelihood (Supplementary Figure S2A) showed that individuals from the same populations were not uniquely clustered. Interestingly, some Cross River samples clustered together with high posterior and bootstrap values. The haplotype network showed that many of the haplotypes of the merged regions have low frequencies (Supplementary Figure S2B). Indeed, very few were found in more than one population. The presence of few population-specific phylogenetic clusters might be attributable to admixture or recent population divergence.

To check the admixture hypothesis, we investigated the population structures of the sampled populations using the five concatenated mitochondrial regions. The overall fixation index for the Nigerian populations was 0.32306. AMOVA (Excoffier et al., 1992) showed that 67.69% of the molecular variance in Nigerian populations was within population, while less than a third of the total variance was among populations (Supplementary Table S3, *p*-value < 10^−5^). The overall exact test of differentiation based on the haplotypes frequencies (Raymond and Rousset, 1995) was also significant (*p*-value < 10^−5^). Pairwise comparisons of the F_ST_ values further showed the existence of population structures across various Nigerian population pairs (Supplementary Table S4). Consistent with the observation in the phylogenetic trees, the pairwise comparison between the Cross River population and other Nigerian populations showed significant F_ST_ values. The pairwise exact test of differentiation based on the haplotype frequencies similarly showed significant values across certain Nigerian populations (Supplementary Table S5).

After establishing the existence of some level of population structure across multiple Nigerian populations, we then proceeded to check the demographic dynamics of the populations. The skyline plots showing the population size dynamics over time revealed that the population size remained stable in Ekiti, Osun and Edo populations (Supplementary Figure S3). Ondo and Cross River populations showed slight recent population expansion but the mismatch distribution (Harpending, 1994) showed that the population size changes were not statistically significant in any of the investigated populations (data not shown). It is important to note that the number of sample sizes for the individuals having the five regions were not enough to estimate demographic history in Oyo and Ogun. These data established the existence of a certain degree of population structure in Nigerian grasscutter populations with little evidence for recent population expansion.

### 3.2 Detailed analyses of the genetic diversity of the Nigerian grasscutter populations with partitioned mitochondrial regions

**FIGURE 3:**
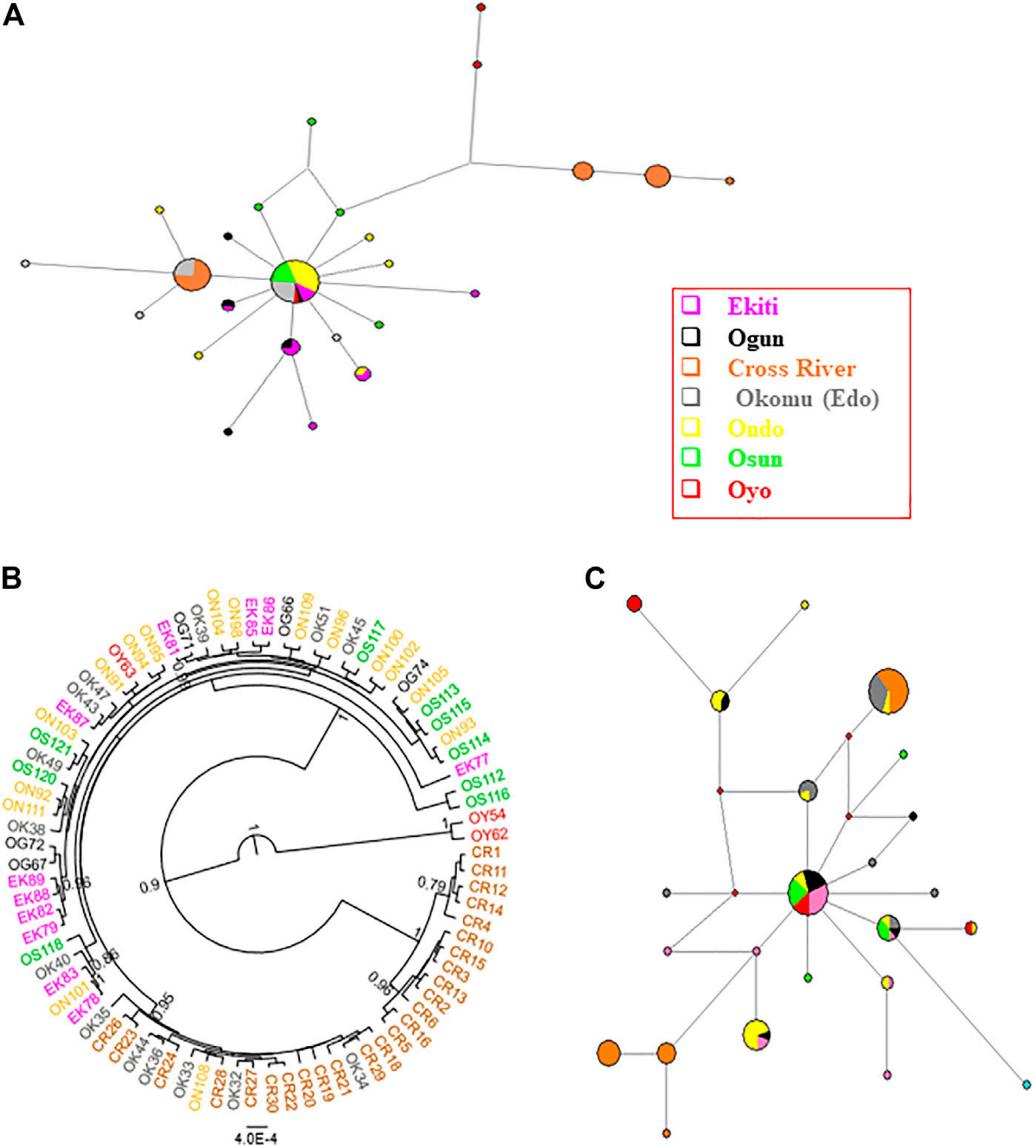
Phylogenetic analyses of Nigerian grasscutter population. The haplotypes are colored according to the sampling locations. **(A)** The haplotype network for the Nigerian grasscutters based on concatenated *CYTB*, *COI*, 16 S and 12 S mitochondrial regions. **(B)** The rooted phylogenetic tree computed from Bayesian inference for Nigerian grasscutter sequences based on the concatenated *CYTB*, *COI*, 16 S and 12 S mitochondrial regions. Branches with at least 0.5 posterior probability are shown. **(C)** The haplotype network for the Nigerian grasscutters based on mitochondrial D-loop region.

Having established the existence of genetic structures across numerous Nigerian populations, we then proceeded to investigate the genetic parameters of the populations. Based on the results of PartitionFinder2, the five mitochondrial regions were partitioned into two schemes, with D-loop being separated and the other four regions forming a cluster. Although the two schemes had a similar evolutionary model, D-loop tended to have higher pairwise distances than other regions (Supplementary Figure S1). We therefore analyzed the D-loop region separately. The complete set of four concatenated mitochondrial regions (*CYTB*, *COI*, 16 S and 12 S) were obtained in 83 Nigerian samples spread across the investigated locations (Figure 1, Supplementary Tables S1, S6). The total length of the four concatenated mitochondrial aligned regions was 1,936 bp, out of which 42 sites were variant. The 83 Nigerian samples were assigned into 26 haplotypes (Figure 3A; Table 1; Supplementary Table S6). The overall haplotype diversity for Nigerian samples was (0.845 ± 0.030). Location-based haplotype diversities ranged from 0.542 (± 0.147) in Ondo subpopulation to 1 (± 0.127) in Ogun and Oyo subpopulations (Table 1). The nucleotide diversities ranged from 0.101 (± 0.052) in Cross River to 0.667 (± 0.523) in Oyo, with the overall nucleotide diversity for Nigerian grasscutters being (0.041 ± 0.022). Tajima’s and Fu’s tests of neutrality showed that the locations with significant values had negative values.

**TABLE 1:** Genetic diversity of Grasscutter populations based on 1,936 bp concatenated mitochondrial regions.

Population	*N*	nHT	HTdiv	πdiv	*D*	*Fs*	SSD	HRI	MNPD
Cross River	26	5	0.70 (0.06)	0.101 (0.052)	−2.435*	15.742	0.142	0.155	16.892 (7.767)
Ekiti	11	6	0.87 (0.071)	0.259 (0.177)	−0.998	−1.464	0.003	0.042	2.073 (1.254)
Ogun	5	5	1.00 (0.127)	0.450 (0.361)	−0.41	−3.304*	0.052	0.24	1.800 (1.236)
Okomu-Edo	14	5	0.703 (0.101)	0.207 (0.156)	−1.227	−1.092	0.017	0.124	1.242 (0.835)
Ondo	16	6	0.542 (0.147)	0.125 (0.099)	−2.110*	−2.677*	0.002	0.067	1.000 (0.710)
Osun	9	5	0.722 (0.159)	0.306 (0.242)	−0.689	−1.995*	0.012	0.11	1.222 (0.854)
Oyo	3	3	1.000 (0.272)	0.667 (0.523)	0	1.272	0.292	0.667	11.333 (7.123)
Nigeria	84	26	0.845 (0.030)	0.041 (0.022)	−2.703*	−2.992	0.025	0.043	7.743 (3.643)
Benin	11	6	0.873 (0.071)	0.242 (0.150)	−1.286	−0.064	0.215	0.08	3.636 (1.994)
Cameroon	24	5	0.638 (0.061)	0.126 (0.092)	−1.770*	−0.293	0.028	0.184	1.257 (0.822)
Ghana	11	9	0.964 (0.051)	0.189 (0.100)	−2.159*	3.486	0.076	0.197	68.691 (32.135)
South Africa	2	1	–	–	–	–	–	–	–

*N* = Total number of samples analyzed; nHT, number of haplotypes; HTdiv, Haplotype diversity, πdiv = Nucleotide diversity; *D* = Tajima’s *D* test of selective neutrality; *Fs* = Fu’s *Fs* test of selective neutrality; SSD, sum of square deviation for mismatch distribution; HRI , Harpending’s raggedness index for mismatch distribution; MNPD, Mean number of pairwise differences. The values in braces are the standard deviations. Asterisks indicate statistical significance at 5% level. Benin = Republic of Benin.

The haplotype network of the concatenated regions showed that the major haplotype observed in about 33% of all the Nigerian samples was not detected in Cross River population (Figure 3A). Interestingly, the second most abundant haplotype, in about 19% of the total samples, was found exclusively in Cross River and Edo populations. In addition, the next two most abundant haplotypes were found exclusively in Cross River samples. The phylogenetic trees with Bayesian inference (Figure 3B) and maximum likelihood method (Supplementary Figure S4) clearly showed the phylogenetic distinctness of some individuals from Cross River and two Oyo samples with high statistical supports. Indeed, F_ST_ values suggest the existence of some level of population structure across Nigerian subpopulations (Table 2). Consistent with the results of F_ST_, the population structure tests using the haplotype frequencies showed that some Nigerian population pairs differed significantly (Supplementary Table S7). The AMOVA revealed the level of population structure across Nigerian subpopulations (Supplementary Table S8). Specifically, 83.59% of the variance component in Nigerian population was within subpopulations, while only 16.41% was among the subpopulations.

**TABLE 2:** Pair-wise difference F_ST_ between subpopulations in Nigeria, Cameroon, Republic of Benin, and Ghana populations based on 1,936 bp of the four concatenated mitochondrial regions.

	Cross river	Edo	Oyo	Ogun	Ekiti	Ondo	Osun	Cameroon	Benin	Ghana
Cross River	-									
Edo	0.121*	-								
Oyo	0.140*	0.718*	-							
Ogun	0.061	0.181*	0.540*	-						
Ekiti	0.127*	0.154*	0.638*	−0.052	-					
Ondo	0.146*	0.085*	0.742*	0.119*	0.075*	-				
Osun	0.095*	0.151*	0.649*	0.115*	0.102*	0.054*	-			
Cameroon	0.177*	0.797*	0.703*	0.785*	0.766*	0.804*	0.778*	-		
Benin	0.062	0.140*	0.500*	0.053	0.103*	0.097*	0.063	0.634*	-	
Ghana	0.153*	0.207*	−0.063	0.063	0.170*	0.225*	0.137*	0.196*	0.125*	-

Numbers with asterisks are statistically significant at *p* < 0.05.

### 3.3 Genetic structure of Nigerian grasscutter populations with specific mitochondrial regions

To maximize the number of individuals that could be analysed (Supplementary Table S1), we focussed on the specific mitochondrial regions. Moreover, D-loop region had higher pairwise genetic distances (Supplementary Figure S1) which might be more useful in highlighting recent population history. We therefore first focused on mitochondrial D-loop locus with a larger sample size (*n* = 105; Supplementary Table S1). We identified 35 varying sites, assigned to 22 haplotypes from 478 bp aligned mitochondrial D-loop regions (Supplementary Table S9). The haplotype diversity ranged from 0.659 (± 0.059) in Cross River to (0.837 ± 0.075) in Ondo (Supplementary Table S10). The nucleotide diversity (π) ranged from (0.214 ± 0.127) in Ondo to 0.551 (± 0.335) in Oyo. Although there is a wide range, Tajima *D* and Fu’s *Fs* tests for neutrality with D-loop regions did not show significant values for most of the wild populations investigated in Nigeria. The only exception was in Ekiti populations with a significant negative value (−2.672). On the contrary, the analyses of the Tajima *D* and Fu’s *Fs* tests on *CYTB* revealed significant negative values for most populations (Supplementary Table S11).

The haplotype network of the Nigerian wild grasscutter populations revealed the distribution of different haplotypes of the D-loop regions (Figure 3C). The two most abundant haplotypes, which represented 43% of the total sampled individuals, had different subpopulation distributions. The most abundant haplotype (*n* = 23 or 22%) was found in Cross River (*n* = 14), Edo (*n* = 8), and to a less extent in Ondo (*n* = 1). The second most abundant haplotype (*n* = 22 or 21%), which seemed to be more central, and more likely to have contributed to the radiation of several other haplotypes is found in all populations except Edo and Cross River populations. Although, low-frequency haplotypes were exclusively restricted to certain populations, several haplotypes occurred in multiple populations. Similar analyses with *CYTB,* 16 S, 12 S and *COI* mitochondrial regions, which were known to contain sites under various levels of purifying selections, revealed different patterns (Supplementary Figure S6A–D). For example, while 22 different haplotypes were found on the D-loop region, the *CYTB* and 16 S regions had only eight haplotypes (Supplementary Figures S6A, B), suggesting higher haplotype diversity in D-loop region. Indeed, 12 S had only four haplotypes (Supplementary Figure S6C), while *COI* had 25 haplotypes (Supplementary Figure S6D). Furthermore, single major haplotypes represented a significant percentage of the sampled individuals in the four mitochondrial regions. For example, 101 of the 108 12 S region belonged to the same haplotype (Supplementary Figure S6C). The haplotype networks of D-loop and *CYTB* were consistent with the observations of the neutrality tests of Tajima’s *D* and Fu’s *Fs* (Supplementary Tables S10, S11). Especially for Tajima’s *D*, most Nigerian populations had significantly negative values for *CYTB*, but the values were not significant for D-loop region (Supplementary Table S10).

Overall analysis of molecular variance (AMOVA) of D-loop for the Nigerian wild grasscutter populations revealed that 86.14% of the population variance were within-population variance (*p*-value < 0.001) (Supplementary Table S12). AMOVA of *CYTB* sequences of Nigerian populations revealed that higher percentage of the variance (92%) was within-population (*p*-value < 0.001) (Supplementary Table S13). The pairwise fixation index (F_ST_) computed from D-loop sequences showed consistency with the geographical locations; Cross River population seemed to be isolated from other populations (Supplementary Table S14). Indeed, most of the population pairs had significant F_ST_ values. However, the pairwise F_ST_ values between Ekiti, Ondo and Ogun populations were not significant. Likewise, the F_ST_ value for Osun and Ogun populations was not significant. The tests of significance of F_ST_ showed that Cross River and Oyo subpopulations were genetically isolated from other wild Nigerian grasscutter subpopulations. Similar results were found when the haplotype frequencies of the populations were compared in a pairwise manner (Supplementary Table S15).

### 3.4 Genetic structure of grasscutter populations across Guinean Forests of West Africa based on the concatenated mitochondrial regions

Having highlighted some of the features of the Nigerian wild grasscutter populations, we then investigated how these features differ across the neighbouring countries in the lower Guinea forests of West Africa. Geographically, Cameroon is close to Cross River (Nigeria), and Republic of Benin is very close to Ogun and Oyo states (Nigeria) (Figure 1). We also retrieved publicly available data from Ghana and South Africa wild grasscutter populations. We first confirmed, with the pairwise distance PCA, that all the samples were of reliable quality. The PCA from the pairwise distances showed that TswiT996 was questionable (Supplementary Figures S7A, B). We therefore excluded the sample from further analysis. Because no individual from Ghana had all the five mitochondrial regions (Supplementary Table S1), we followed the data partition scheme and separately analysed the concatenated four regions (*COI*, *CYTB*, 16 S and 12 S). Note that the D-loop region was analyzed separately. The aligned sequences of the four concatenated regions were 1,974 bp long and included 131 grasscutter samples from Nigeria, Cameroon, Republic of Benin, Ghana, and South Africa. These aligned sequences with 276 variant sites were assigned to 45 haplotypes (Supplementary Table S6). The haplotype diversity ranged from 0.638 (± 0.061) in Cameroon to 0.964 (± 0.051) in Ghana (Table 1). The nucleotide diversity ranged from 0.041 (± 0.022) in Nigeria to 0.242 (± 0.150) in Republic of Benin. Whereas the Fu’s statistic was not significant, Tajima *D* was significantly negative in Nigeria, Cameroon, and Ghana grasscutter populations. Indeed, the skyline plots support the possibility of recent population expansions in Nigeria and Cameroon, but not for the Ghana population (Supplementary Figure S8). However, Harpending’s raggedness index and sum of square deviation (SDD) were not significant (Table 1).

**FIGURE 4:**
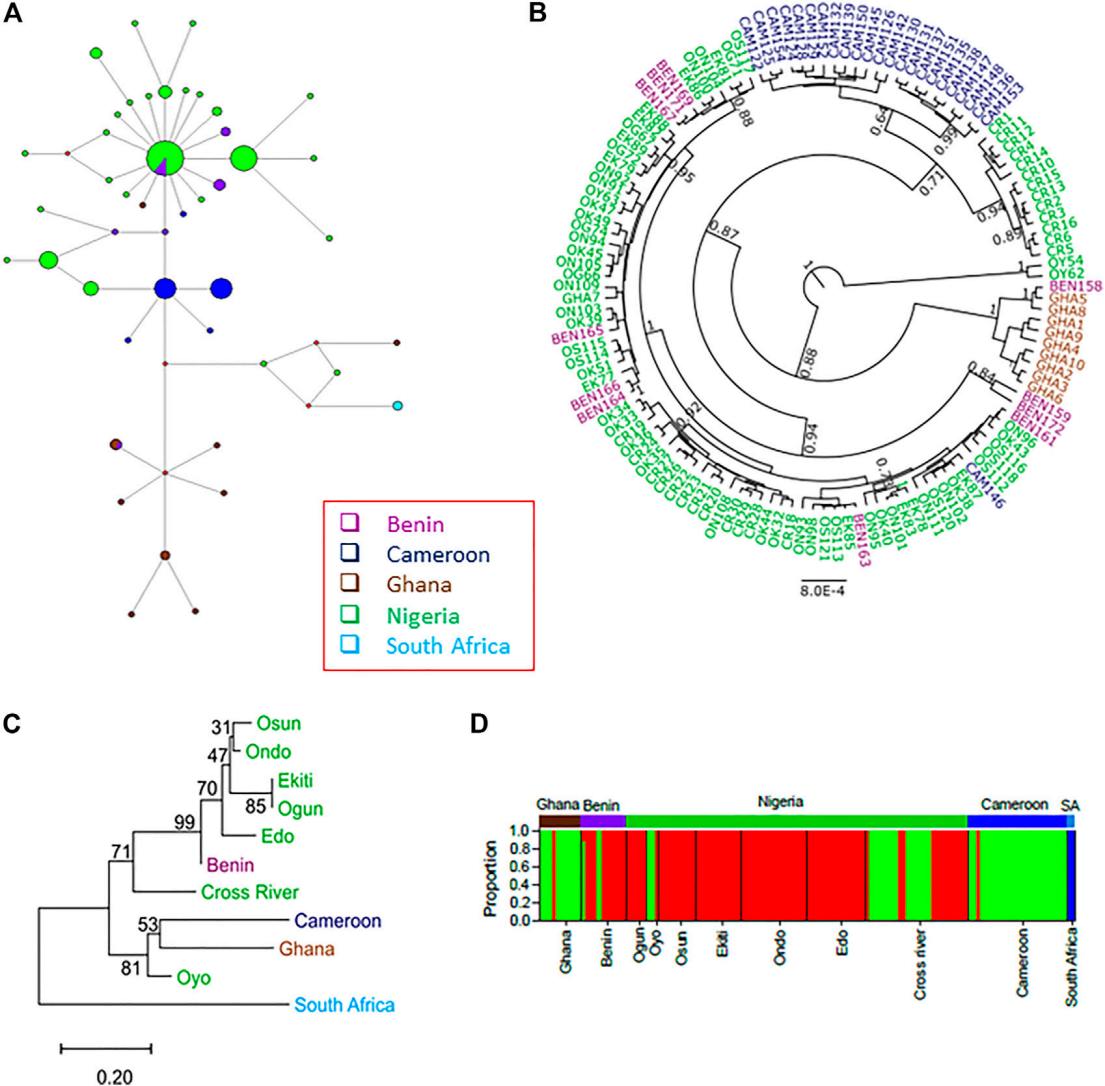
The population structures of the wild grasscutters from the Guinean Forests of West Africa based on the concatenated *CYTB*, *COI*, 16 S and 12 S mitochondrial regions. **(A)** The haplotype network for the sampled individuals. **(B)** Phylogenetic tree computed from the Bayesian inference of the grasscutter sequences. Branches with at least 0.5 posterior probability are shown. **(C)** The population tree computed from the corrected F_ST_ values. The bootstrap values from 1,000 replications are shown. **(D)** Structure analysis to reveal the genetic components of various grasscutter populations. The optimum number of component (k) was three. Panels **(A–D)** were made from the concatenated *CYTB*, *COI*, 16 S and 12 S mitochondrial sequences.

The network based on the four concatenated mitochondrial regions revealed that many of the haplotypes were country-specific (Figure 4A; Supplementary Table S6). Only two haplotypes were shared between countries. One of the haplotypes (*n* = 31) was shared between Nigerian and Republic of Benin populations, while the other (*n* = 3) was shared between Ghana and Republic of Benin samples. This suggested the existence of population structures across countries. The phylogenetic analyses using Bayesian inference (Figure 4B) and maximum likelihood (Supplementary Figure S9) revealed that haplotypes from the same country tended to cluster together. Indeed, pairwise F_ST_ was significant across each pair of countries (Table 2). The only exception was between Nigeria and Republic of Benin where the F_ST_ was not significant. Generally, the geographical distance between the populations correlated (Pearson’s r = 0.58, *p*-value = 3.3e-05) with the genetic distance between the populations (Supplementary Figure S10A). The test of population structure using the haplotype frequencies (Supplementary Table S7) also showed similar patterns to the results of F_ST_ (Table 2). Again, the haplotype frequencies of some Nigerian subpopulations and population from the Republic of Benin were not significantly different. AMOVA for the concatenated sequences revealed the status of the population structure (Supplementary Table S8). For all the samples from the investigated populations, about 73% of the overall total variation was among population while about 27% was within population.

Population phylogenetic tree using the F_ST_ values computed from the four mitochondrial regions reveal the relationships among the populations. For clearer understanding of the phylogenetic relationship, each of the seven Nigerian populations was treated individually. The result showed that the South Africa population diverged first from the other populations (Figure 4C). Surprisingly, Oyo population was found to cluster with both Ghana and Cameroon populations. Consistent with the network in Figure 4A, Benin population was found to cluster with other Nigerian populations.

To better understand the population phylogenetic tree, we investigated the genetic components of each of the populations. We investigated various numbers of ancestral genetic components (k = 2–11) for models with or without admixture, and model involving migration (Supplementary Figure S11). At all the investigated k values in models without migration, the South African components were not found in any other individuals, consistent with the results of the phylogenetic analyses. However, for the model involving migration at k = 2, some Ghana and Oyo individuals showed some levels of South African components. Interestingly, at k = 3 and above, the genetic components did not substantially change in the models without migration. However, some differences were observed across different k values in the model involving migration. Another obvious difference across the models was the number of admixture events. More admixture events were found in the model involving migrations, while the model without admixture expectedly had the least. Interestingly, admixture was found for some individuals from the Republic of Benin at k > 2, even for the model without admixture (Supplementary Figure S11).

In any case, we proceeded to investigate the “best” k value for the grasscutter populations. For all the k values, the STRUCTURE’s choice criterion was higher for models without migration (Supplementary Figure S12A). The models without migration reached the plateau at k = 3, while the migration model had a peak at k = 4. The model choice method involving ΔK (Evanno et al., 2005) confirmed these k values (Supplementary Figure S12B). Figure 4D showed the distribution of ancestral components in each of the populations at k = 3 for the model without admixture and migration. As expected, South African component was not found in any other population. Consistent with the population phylogenetic tree, the dominant component in Nigerian populations was the minor component in both Ghana and Cameroon populations. Oyo and Cross River populations had some levels of Ghana and Cameroon component. Although the majority of the Benin population had the Nigeria component, some individuals had Ghana and Cameroon components.

Because of the limited number of loci and STRUCTURE’s assumptions, we used DAPC to further investigate the genetic components in the populations studied. Consistent with the cluster results for models without migration, South African samples were separated from other populations at all k values tested (Supplementary Figure S13). The results for k = 2 and k = 3 were very similar for the DAPC results and the STRUCTURE’s models without migration (Supplementary Figures S11, S13). The genetic components observed for Oyo, Cross River, and Benin in DAPC were similar to the results in STRUCTURE without migration. At higher k values, DAPC was able to capture higher levels of differentiation. At all the k values investigated, samples from the same location tended to have more similar genetic components. Also, samples from the Republic of Benin had similar genetic components to some Nigerian samples. Unlike the results of STRUCTURE, especially when migration model was considered, DAPC did not find multiple signals of admixture.

### 3.5 Analyses of specific regions reveal more detailed dynamics of grasscutter populations across Guinean Forests of West Africa

We next analysed the D-loop sequences from the countries. A total of 234 analysed grasscutter mitochondrial D-loop sequences from Nigeria (*n* = 104), Republic of Benin (*n* = 15), Cameroon (*n* = 31) and Ghana (*n* = 84) were assigned to 60 haplotypes (Supplementary Table S9). Unlike in the concatenated regions, the lowest haplotype diversity of D-loop region was found in the Republic of Benin population (0.571 ± 0.149), while the highest in the Ghana population (0.921 ± 0.013) (Supplementary Table S10). Nigerian, Republic of Benin, and Cameroon populations showed statistically significant negative Tajima’s *D* values, suggesting population expansions. Indeed, the skyline plots showed evidence of slight recent population expansions in all the populations but the pattern in Ghana was strange with wide confidence interval in the recent years (Supplementary Figure S8). In addition, analyses of *CYTB* revealed similarly lower haplotype and nucleotide diversity in Republic of Benin populations (Supplementary Table S11). However, only Nigerian and Republic of Benin populations showed significantly negative Tajima’s *D* values for the *CYTB* region.

**FIGURE 5:**
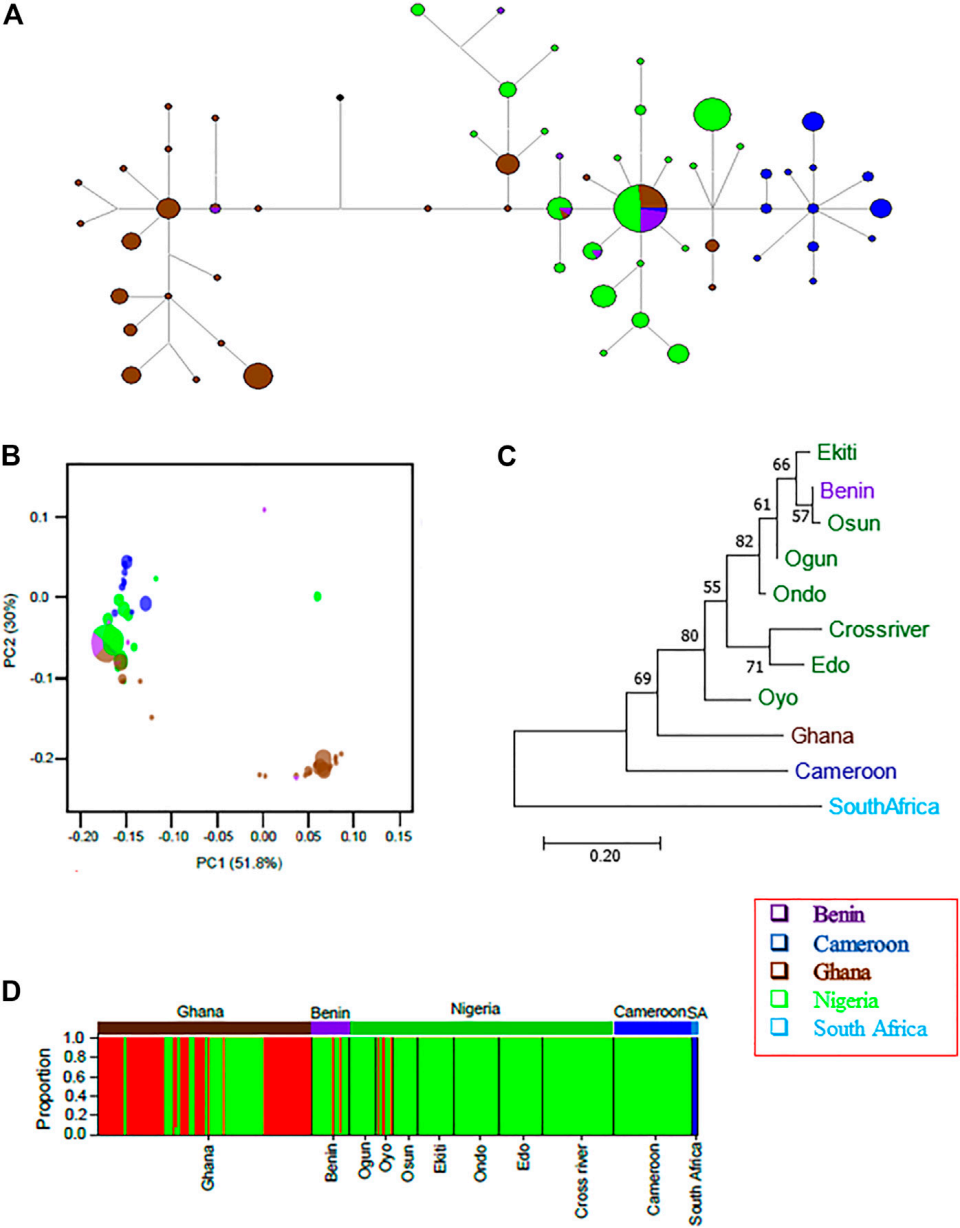
Mitochondrial D-loop regions reveal detailed population structure of wild grasscutter populations from Guinean Forests of West Africa. **(A)** The haplotype network for the grasscutters based on mitochondrial D-loop region. **(B)** PCA computed from pairwise genetic distances showing the relationships between various populations. D-loop haplotypes were used for the computation. The colors correspond to the country of sampling while the size is proportional to the haplotype frequency. **(C)** The population phylogenetic tree based on the corrected F_ST_ computed from the D-loop regions. **(D)** Structure analysis to reveal the genetic components of various grasscutter populations. The optimum number of component (k) was three. Panels **(A–D)** were made from the mitochondrial D-loop sequences.

Although there were some exceptions, the haplotype network for the D-loop regions closely mirrored the geographical locations of the haplotypes (Figure 5A), and was largely consistent with the concatenated mitochondrial region result (Figures 4A, B; Supplementary Figure S9). The most abundant D-loop haplotype (*n* = 45 or 19%) was found in the four countries under investigation. However, many of the haplotypes in the D-loop regions were specific to each country. The analyses of *CYTB*, *COI*, 12 S and 16 S regions revealed patterns expected under purifying selections or selective sweep with fewer haplotypes dominated by numerous low-frequency haplotypes (Supplementary Figures S14A–D), largely reflecting the results of Nigerian subpopulations. Interestingly, the *CYTB* haplotype with the highest frequency in Nigeria populations (68%), Republic of Benin population (93%) and Cameroon population (55%) are not found in Ghana (Supplementary Figure S14A), suggesting the isolation of Ghana populations.

Despite the haplotype sharing between Republic of Benin and Nigerian populations, the D-loop network showed clear pattern of population structure (Figure 5A), and the AMOVA revealed that 58.98% of the variance was within populations (Supplementary Table S12). This level of within-population variance was lower than the value for Nigerian populations. Consistent with the haplotype network, significant genetic structure was found in all pairs of the populations across countries (Table 2, Supplementary Figure S15). As expected from the geographical distribution, the highest F_ST_ value (0.516) was found between Ghana and Cameroon populations, while the lowest F_ST_ value (0.042) was found between Nigerian and Republic of Benin populations. Indeed, the correlation between the geographical distance and the genetic distance was higher in D-loop region (Person’s r = 0.75, *p*-value = 4.9e-09) than in the merged regions (Supplementary Figure S10B). The pairwise tests using the haplotype frequencies (Supplementary Tables S15) produced similar results to F_ST_ tests.

Principal component analyses were then computed using pairwise genetic distances of the D-loop haplotype sequences. PC1, which accounted for about 52% of the variance separate a cluster of Ghanian haplotypes from others (Figure 5B). Also, Cameroonian population seemed to form a cluster that was not too far separated from the Nigerian and Republic of Benin populations, suggesting more recent shared ancestry. To better visualize the relationship, we computed the population phylogenetic tree using the D-loop sequences (Figure 5C). The result confirmed that South African population was the outgroup. Among the other populations, Cameroon population diverged first before the Ghana population diverged from the Nigerian population. Again, Benin population was found to cluster with Nigerian populations. Pairwise F_ST_ analyses using *CYTB* (Supplementary Table S16) revealed essentially similar patterns as the D-loop (Supplementary Table S14). Despite the small sample sizes for South African (*n* = 3) and Equatorial Guinea (*n* = 2) populations, the signatures of populations structures were still revealed.

We next checked the ancestral genetic components for the populations based on the D-loop sequences. We checked the components for k = 2 to k = 11 using three different models in STRUCTURE software (Supplementary Figure S16). Consistent with phylogenetic results and the results of the four merged regions, for all the investigated k values, South African samples were consistently separated from the other populations and the genetic components of most of the individuals from the Republic of Benin were found in some Nigerian individuals. Like in the merged regions, more admixture events were found in the model involving migration. Also, the STRUCTURE results were mostly similar for k = 3 and above in both admixture model and the model without admixture. The model involving migration revealed more structures at higher k values. Both STRUCTURE choice criterion (Supplementary Figure S12C) and ΔK (Supplementary Figure S12D) showed that k = 3 best fit our data for models with or without admixture. The best k value for the model involving migration was difficult to resolve (Supplementary Figure S12D). At k = 3 (Figure 5D), Cameroon and Benin components looked more similar to Nigerian component while the Ghana population was more heterogenous. Some individuals in Oyo population also contained different genetic component.

We next repeated the analyses of the D-loop region using DAPC. Like for the STRUCTURE results, the South African samples were consistently shown to be genetically different at all k values investigated (Supplementary Figure S13). The results of DAPC for k = 2 and k = 3 mostly reflected the results of STRUCTURE analyses for models without migration. At higher k values, DAPC revealed more population structures. In all the k values investigated, the population from Benin Republic had more similar genetic components to some Nigerian individuals. At k = 4, four components from South Africa, Cameroon, Nigeria/Benin and Ghana populations were seen. However, at all k values investigated, the analyses of the D-loop showed that some individuals from Ghana have Nigeria/Benin genetic components.

### 3.6 Phylogenetic analysis and evolutionary history of grasscutter populations based on *CYTB* region

**FIGURE 6:**
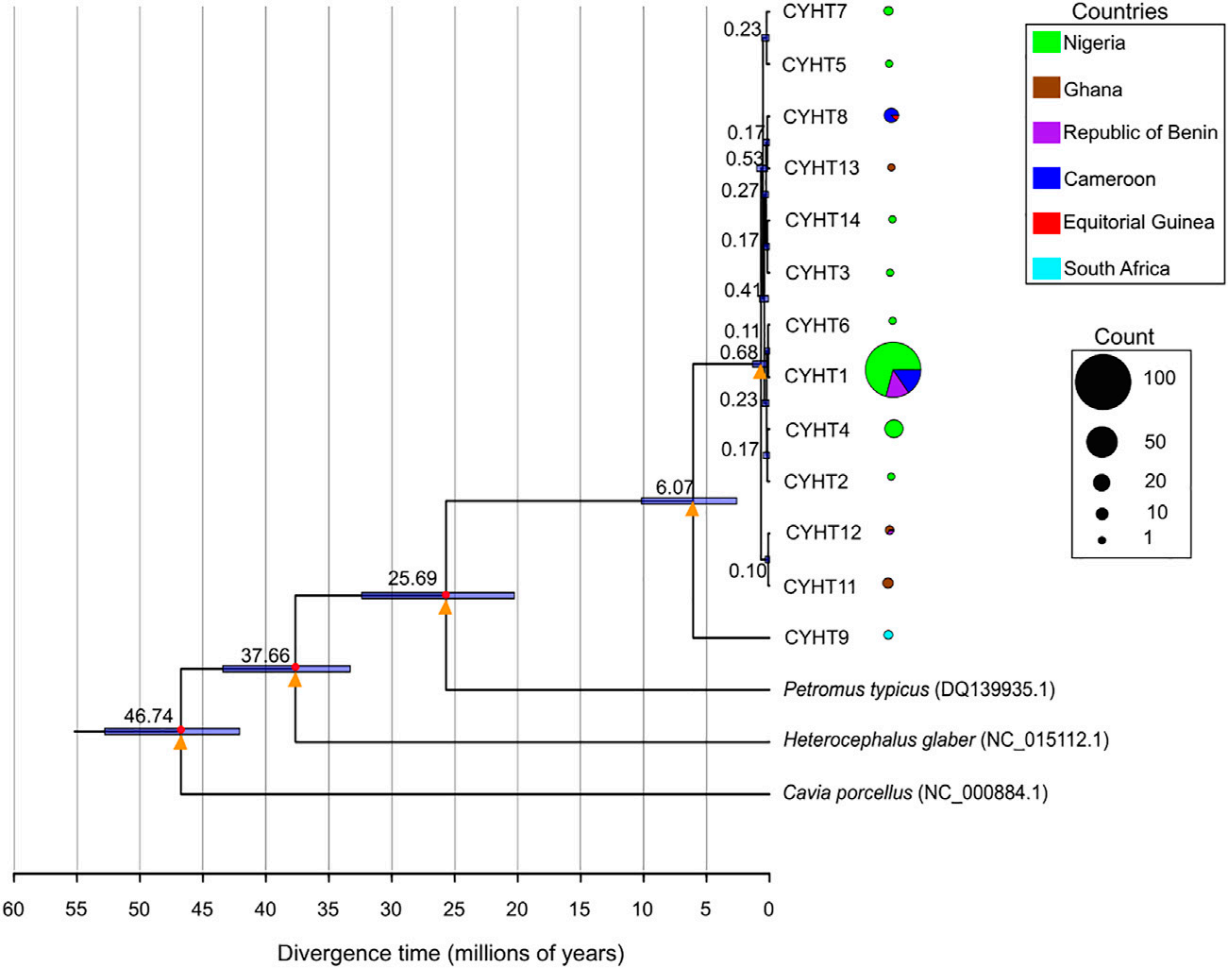
Divergence time estimates of the *CYTB* haplotypes. The divergence time estimates are shown in each node. The bars represent 95% of the estimates for the nodes. The calibration points are marked with red circles. The yellow triangles represent nodes with very high statistical supports (posterior probability = 1, bootstrap support value from maximum likelihood method with 1,000 replicate > 95%). The distribution of each haplotype is presented in pie chart, with the size proportional to the total number of the haplotype.

Apart from lesser cane rat, which is in the same genus as grasscutter, the next closest species was dassie rat (*P. typicus*). However, there are no nucleotide sequences of any of the five studied mitochondrial regions for lesser cane rats, and there was paucity of the sequences of mitochondrial regions for dassie rats. Thus, we restricted the divergence time estimate to *CYTB* region with dassie rat sequence (Visser et al., 2019). The *CYTB* sequences formed 13 haplotypes across the investigated countries (Supplementary Table S17). The phylogenetic analyses revealed that the haplotype exclusively found in South African was separated from other grasscutter *CYTB* haplotypes (Figure 6; Supplementary Figure S12A). The South African *CYTB* haplotype was estimated to have diverged from other haplotypes at about 6.07 (2.6–10.18, 95% CI) MYA. Although only three samples from South African were analysed, the fact that the haplotype was observed in none of the 234 West African and Cameroonian individuals (Figure 6), despite the high level of *CYTB* haplotype sharing among West African and Cameroonian populations (Supplementary Figure S12A), suggested an ancient separation of South African population from the other studied populations.

The haplotypes from Nigeria, Cameroon, Republic of Benin, and Ghana shared a much more recent history with the last common ancestor diverging about 0.68 (0.23–1.341, 95% CI) MYA. The phylogenetic relationship of the *CYTB* haplotypes revealed that after the divergence of the South African haplotype, the next haplotypes to diverge were found in Ghana and Republic of Benin. Importantly, one of the Ghanaian haplotypes was also shared by the Republic of Benin population. The sharing of *CYTB* haplotype between Equatorial Guinean and Cameroonian populations was also worthy of note. The phylogenetic analyses revealed that relatively recent emergence of the major *CYTB* haplotype. Because *CYTB* experienced purifying selection, it was expected that many shared haplotypes would still be present even after a long evolutionary period. The relaxed evolutionary pressure in D-loop regions would lead to gradual decrease of shared haplotypes. Taken together, the mitochondrial D-loop and *CYTB* regions presented a more comprehensive picture of the population structure and migration history of wild grasscutter populations across lower Guinean forests.

## 4 Discussion

This study investigates the population dynamics of Nigerian wild grasscutter population with populations from other African Guinea forest countries using the nucleotide sequences from five mitochondrial regions. While the *CYTB,* 16 S, 12 S and *CO1* are likely to show incomplete lineage sorting, D-loop tends to reveal more recent evolutionary history. Consistent with functional importance, the D-loop region reveals more population dynamics and history, potentially because of the relaxed purifying selection. The overall D-loop haplotype diversity for Nigerian grasscutter population (0.912 ± 0.015) is slightly lower than 1.000 (± 0.016) reported for Nigerian cattle (Mauki et al., 2021), but slightly higher than 0.899 (± 0.148) for sheep (Agaviezor et al., 2012), higher than 0.693 (± 0.022) reported for helmeted guinea fowl (Adeola et al., 2015) and 0.673 (± 0.002) for Nigerian local chicken (Lasagna et al., 2020), suggesting the domestication in the species might have lowered the genetic diversity as previously reported in horses and dogs (Wang et al., 2013; Wang et al., 2015; Fages et al., 2019). Importantly, the high genetic diversity of the wild grasscutter populations suggests that there is no loss of genetic resources. Both haplotype and nucleotide diversities revealed differences across the investigated populations from the African Guinea forests. This information could be useful in both conservation efforts and breeding programs.

The analyses of demographic histories reveals the recent population dynamics of the wild grasscutter populations. Whereas significant values of Fu’s *Fs* and Tajima’s *D* could reveal recent population size change, they are originally tests for neutrality which could reflect signatures of selections and/or selective sweeps (Tajima, 1989; Harpending, 1994; Fu, 1997). This suggests that the significant values for these parameters might not necessarily reflect population size changes. Indeed, BICEPS results show that the time to the most recent common ancestor (in substitution unit) tends to be different between the four merged regions and the D-loop, reflecting the impacts of evolutionary constraints. As different regions tend to have different evolutionary constraints different degrees of isolation revealed by different markers might reflect deviation from neutrality. Further, the two parameters rely on infinite site model which might not be adequate for mitochondrial genome. Analyses such as mismatch distribution (Harpending, 1994) and Bayesian approach also establish population changes. It is important to point out that BICEPS (Bouckaert, 2022) assumes random mating with no admixture. Therefore, some subtle signals might be missing because of the nature of our data and the colony structures of grasscutters (Hoffmann, 2008; Coker et al., 2017). Notwithstanding, all the analyses reveal isolated instances of recent population expansion. No signature of population contraction exists, indicating that the wild grasscutter populations are not under threat of extinction. This highlights the classification of the species as “least-threatened” despite the high intensity of hunting.

The determination of the exact number of genetic components in a population or group of populations remains a Herculean task (Pritchard et al., 2000; Evanno et al., 2005). We use two different methods to investigate the best k value under different models in STRUCTURE software. Also, because of the limited number of loci and high possibility that our data might violate some of the assumptions of STRUCTURE, we confirm the STRUCTURE results with DAPC. While the results are essentially similar at lower value, DAPC reveals more structure at higher k values, reflecting the limitation of STRUCTURE models at higher k values, especially when no migration was considered. Whether the additional components detected at higher k values for DAPC and the model involving migration in STRUCTURE are actually genuine or noisy signals could not be ascertained. However, both STRUCTURE and DAPC support our main conclusions.

Although there is high within-population variance and Nigerian populations tend to have the same genetic component, our analyses reveal some level of genetic structures among the Nigerian populations. This is more pronounced when populations are compared across countries. Shared haplotypes are few, especially at the mostly neutrally evolving D-loop locus. The strength of genetic structure relates to geographical distance. The high within-population variance could reflect recent population divergence or admixture, and thus geographical distance. Indeed, a significant positive relationship occurs between the genetic and geographical distances of the populations. However, our data cannot delineate between the recent divergence or admixture hypotheses. Moreover, the genetic distinctness of Cross River subpopulations among the Nigerian grasscutters may be attributable to the elevation of Cross River state, thereby limiting migration. However, the population structure might not be entirely due to migratory limitations as grasscutter have relatively good migratory ability as they can run on land and swim across rivers. One factor that might contribute to the population structure is the colony system (Coker et al., 2017; Mustapha et al., 2020; Kilwanila et al., 2021). Each grasscutter colony comprises a male and several females, thereby creating a form of isolation or structure (Hoffmann, 2008). Owusu et al. (2010) reported decreasing male proportion as litter size increased for grasscutters farms in Ghana. A study of bush meats in Ogun State, Nigeria (Yisau et al., 2019) showed that only 24% of captured juvenile and 40% of sub-adult grasscutter were males, suggesting that the wild litter size ratio might be biased towards females. However, about 61% of captured adults were males. Since most colonies have fewer adult males than females, the colony survival after the death of the breeding male would depend on the taking over by another male from another colony or the replacement by a young male. The replacement by a younger male from the colony would lead to stronger signatures of genetic structure based on maternally inherited mitochondrial DNA.

Although grasscutter is believed to have evolved in Africa (Baptist and Mensah, 1986b; López-Antoñanzas et al., 2004; Hoffmann, 2008), the exact location of the first emergence is not known, and the grasscutter studies have been reported to be biased (Kilwanila et al., 2021). Our results indicate that South African and West African *CYTB* haplotypes diverged at least 6.1 MYA. It is possible that the haplotype divergence may predate population divergence (Maddison, 1997; Suh et al., 2015; Shen et al., 2017). Indeed, the emergence of crown Thryonomidae (the common ancestor of cane rats) has been contested (Kraatz et al., 2013; Sallam and Seiffert, 2016; Sallam and Seiffert, 2020). The inconsistent fossil records and the limited nucleotide sequences for the closely related species restrict fossil calibration to the outgroup species. This could affect the Bayesian estimation of the divergence times. However, the South African samples do not cluster phylogenetically with the samples from other regions in all the investigated genomic regions, suggesting that the divergence is ancient. Although the timing cannot be established with high certainty, the analyses of *CYTB* sequence suggest that Ghana population diverged before the Cameroon population diverged from the Nigerian and Republic of Benin populations. However, the population divergence tree based on D-loop suggests that the Cameroon population diverged first before the split of Ghana and Nigerian/Benin populations. This suggests that *CYTB* might reflect more of gene tree than the population tree. Further, the impacts of purifying selection on the divergence time estimation cannot be fully ascertained. The analyses of nuclear data of different mammalian orders reveal that the use of sites under purifying selection gave more consistent results (Babarinde and Saitou, 2020). However, whether this holds in mitochondrial genomes, and especially in recently diverged population-level individuals is not clear. In any case, our data consistently show that Nigerian and Republic of Benin populations share much more recent history, and that South African population shares a very deep coalescence with other populations.

Grasscutter has been described as a potential livestock for the future (Opara, 2010; Adu et al., 2017). Several efforts are being made in domesticating grasscutters to produce meat for humans (Jori et al., 1995; Adu et al., 1999; Adu et al., 2000; Fayenuwo and Akande, 2002; Adu et al., 2017). Indeed, the meat of the grasscutter is well accepted in West Africa (Aluko et al., 2015; Gaubert et al., 2015; Teye et al., 2020). A classical method of improvement in animal breeding is by heterosis (Birchler et al., 2006; Timberlake, 2013; Akeberegn et al., 2019). Breeding individuals from similar genetic backgrounds can lead to inbreeding depression (Kardos et al., 2016; Hajduk et al., 2018; Kardos et al., 2018; Harrisson et al., 2019). Our study reveals that South African grasscutter crossbred with grasscutters from West Africa would benefit more from heterosis because of their different genetic backgrounds. On the other hand, Republic of Benin and Nigerian populations are not so different genetically. Further, the Republic of Benin population seems to have lower genetic diversity, probably because of the small size of the country. Gain from heterosis with this population would be minimal. Therefore, grasscutter breeding stocks from populations in other countries, outside Republic of Benin, are recommended for grasscutter breed improvement. The second implication of this study is in the understanding of the status of grasscutter populations. This study confirms that the grasscutter populations are generally not threatened.

Although this study reveals some important status of the wild grasscutter populations from the Guinea Forests of West Africa, there are certain limitations of the study. First, the number of individuals that could be analysed is few in some populations. Indeed, the description of the wild grasscutter populations from Equatorial Guinea and South African cannot be extensively investigated because of the extremely low sample sizes. How representative these sampled individuals are for their respective populations cannot be ascertained. Second, the number of informative sites was small. Although the effects of these two factors could be minimized by the number of replications or samplings, the results are still not completely free from stochasticity. Therefore, further studies involving larger sample sizes are recommended for these populations. Finally, this study uses maternally inherited mitochondrial regions with limited number of informative sites, and some analyses that assume infinite site models might not be reliable. Future studies to further confirm the results should be based on nuclear regions sampled from sufficiently large number of individuals from across the locations.

## Data Availability

The data presented in the study are deposited in the NCBI repository with accession numbers MZ418538-MZ418687; MZ418390-MZ418537; MZ418252-MZ418389; MZ418839-MZ418996; MZ418688-MZ418838 for D-loop, cytochrome b (*CYTB*), cytochrome c oxidase I (*COI*), ribosomal subunits 12S and 16S, respectively.
