# Supplementary material for: Population structure and evolutionary history of the greater cane rat (Thryonomys swinderianus) from the Guinean Forests of West Africa
Source: Front Genet. 2023 Feb 27;14:1041103. doi: 10.3389/fgene.2023.1041103 (PMC10010571; doi:10.3389/fgene.2023.1041103)
Supplement: Supplementary file 1 [file Presentation1.pdf]

Supplementary Material

1 Supplementary Data

Sequences are deposited in GenBank under accessions no. MZ418538 - MZ418687; MZ418390 - MZ418537; MZ418252 - MZ418389; MZ418839 - MZ418996; MZ418688 - MZ418838 for D-loop, cytochrome b (*CYTB*), cytochrome c oxidase I (*COI*), ribosomal subunits 12S and 16S, respectively.

2 Supplementary Figures and Tables

Supplementary figures S1-S16 and supplementary tables S2-S17 are presented in this document. Supplementary table S1 is in another document.

2.1 Supplementary Figures

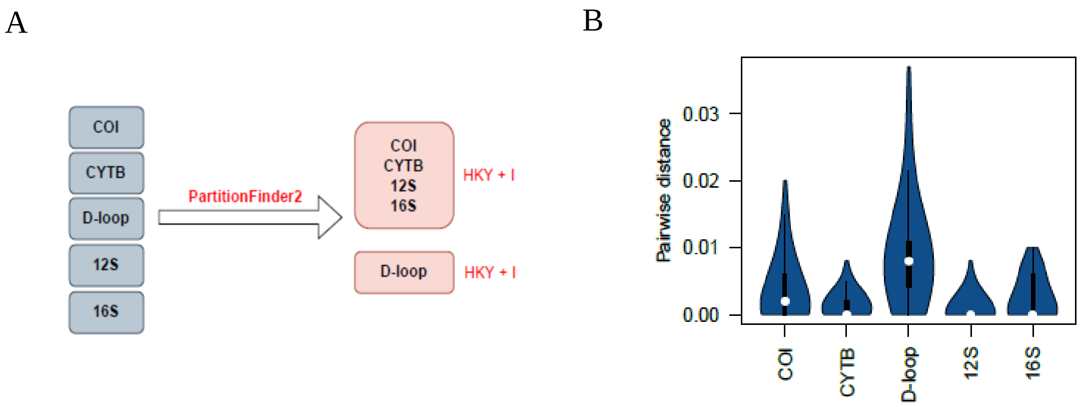

Figure S1. The evolutionary models of the genetic markers used. (A) The partitioning schemes of the markers based on the results of Partitionfinder2. The regions were partitioned into two. The best model for the two schemes was HKY + I. (B) Pairwise genetic distances of *COI*, *CYTB*, D-loop, 12S and 16S mitochondrial regions. The pairwise distances were computed using maximum likelihood composite in MEGA7 (Kumar et al, 2016). Mitochondrial D-loop region had higher genetic distance than other mitochondrial regions.

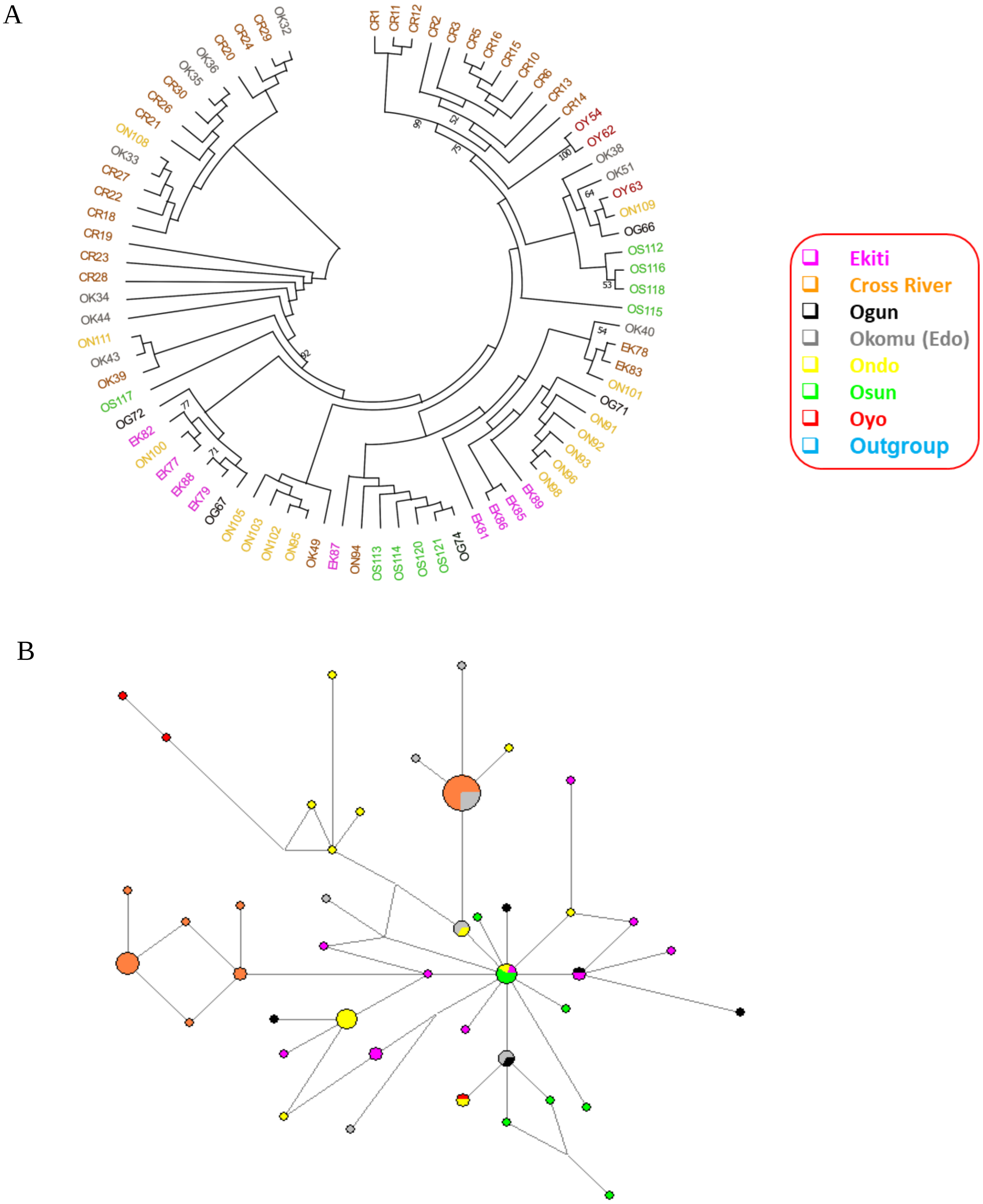

Figure S2. The phylogenetic relationships for Nigerian samples based on the 2,437 bp of the five concatenated mitochondrial regions. (A) Maximum likelihood (ML) tree for the samples. The best tree from 1000 bootstraps is presented. Branches with at least 50% bootstrap support are shown. (B) The haplotype network for the samples. The samples are colored by the sampling location.

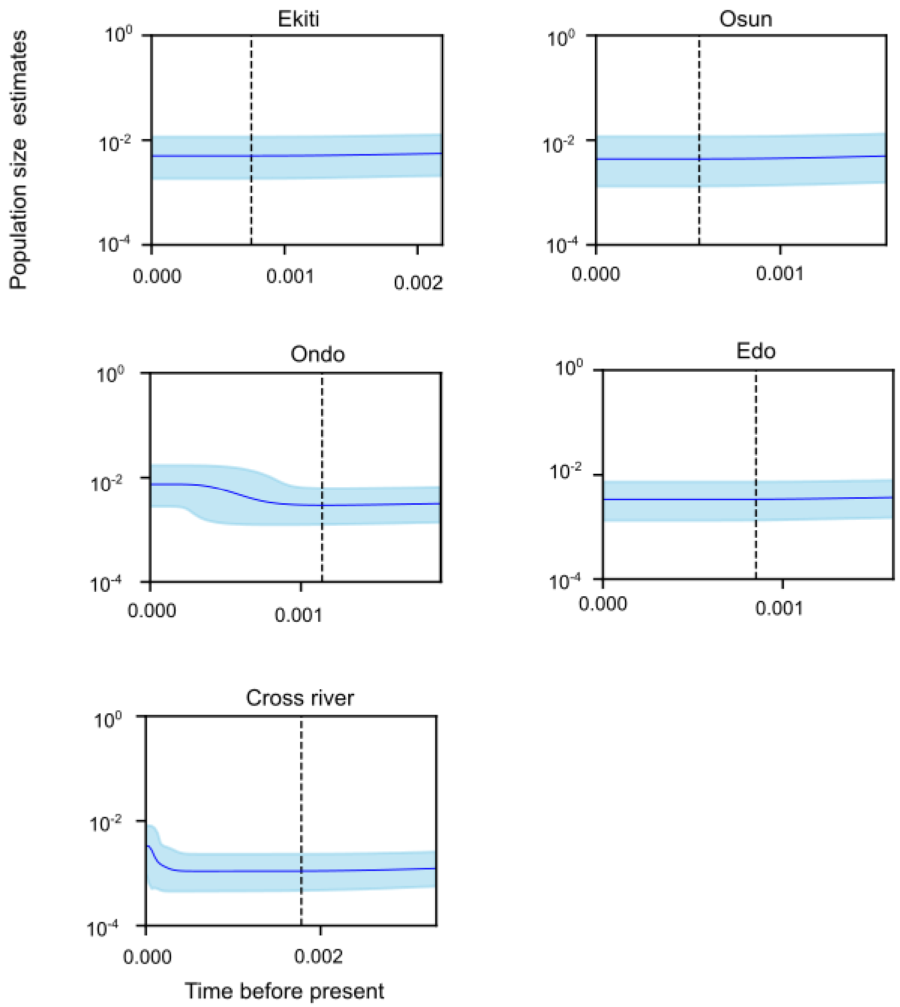

Figure S3. The skyline plots showing the demographic histories of five Nigerian populations based on the 2,437 bp of the five concatenated mitochondrial regions. The population size estimates are shown on the vertical axes while the times before present, in substitution rate units, are shown on the horizontal axes.

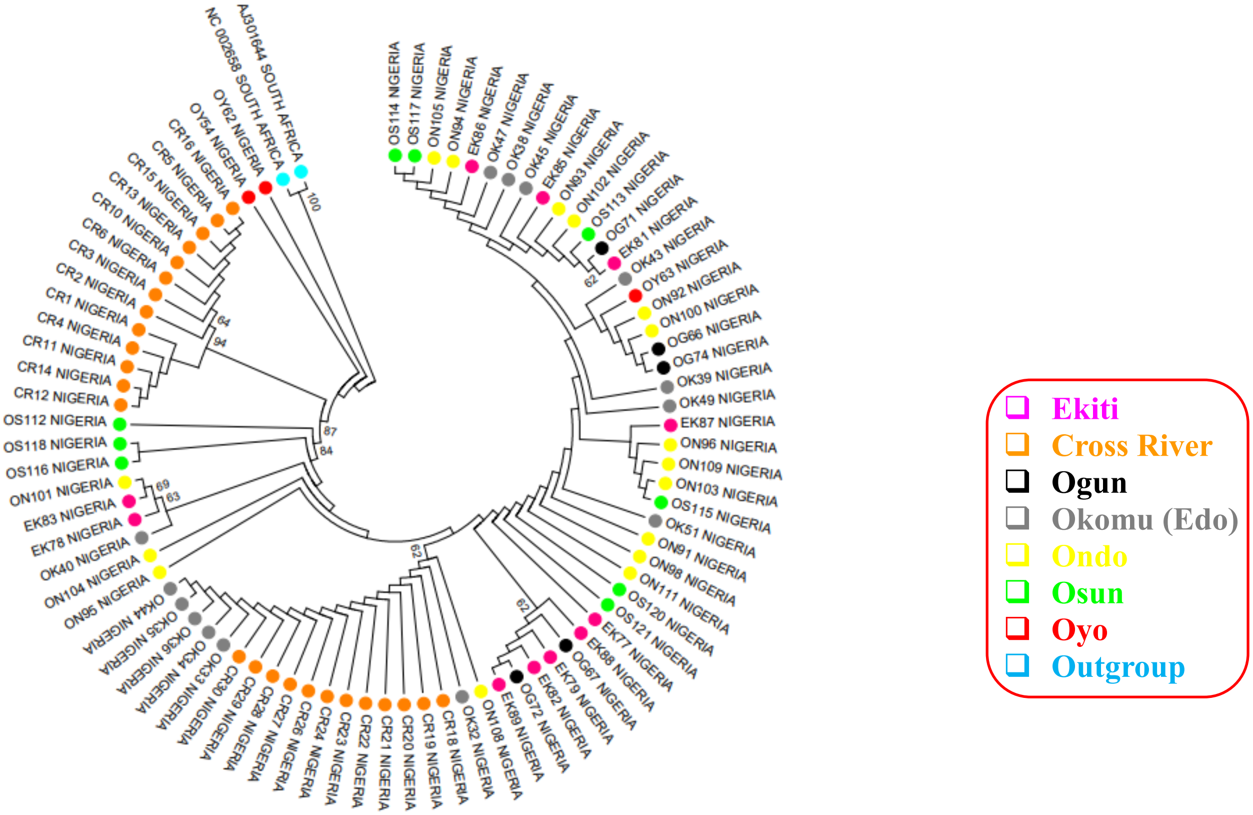

Figure S4. Maximum likelihood (ML) tree for Nigerian and South African samples based on the 1,936 bp of four concatenated mitochondrial regions. The samples are colored by the sampling location. The best tree from 1000 bootstraps is presented. Branches with bootstrap support of 60% are shown.

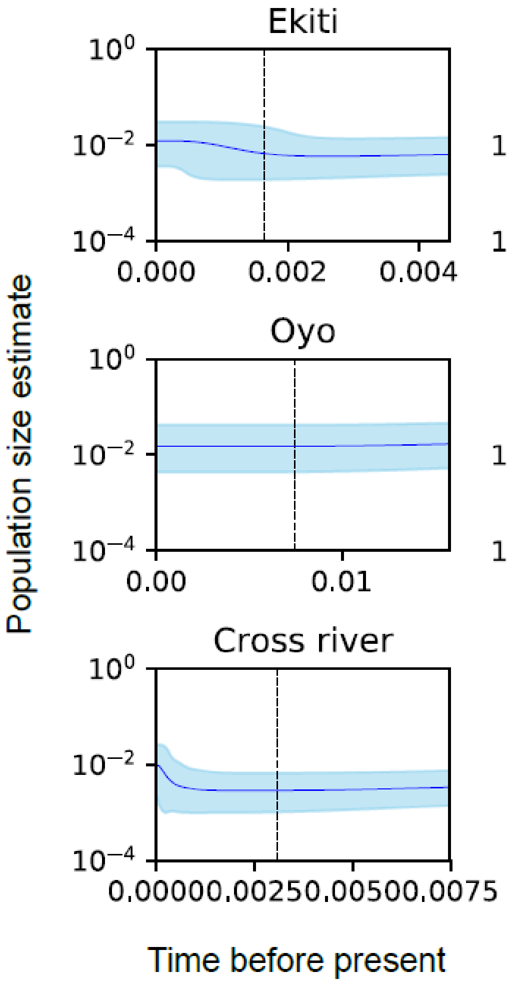

Figure S5. The skyline plots showing the demographic histories of Nigerian populations based on the mitochondrial D-loop region. The population size estimates are shown on the vertical axes while the time, in substitution per site unit, is shown on the horizontal axes.

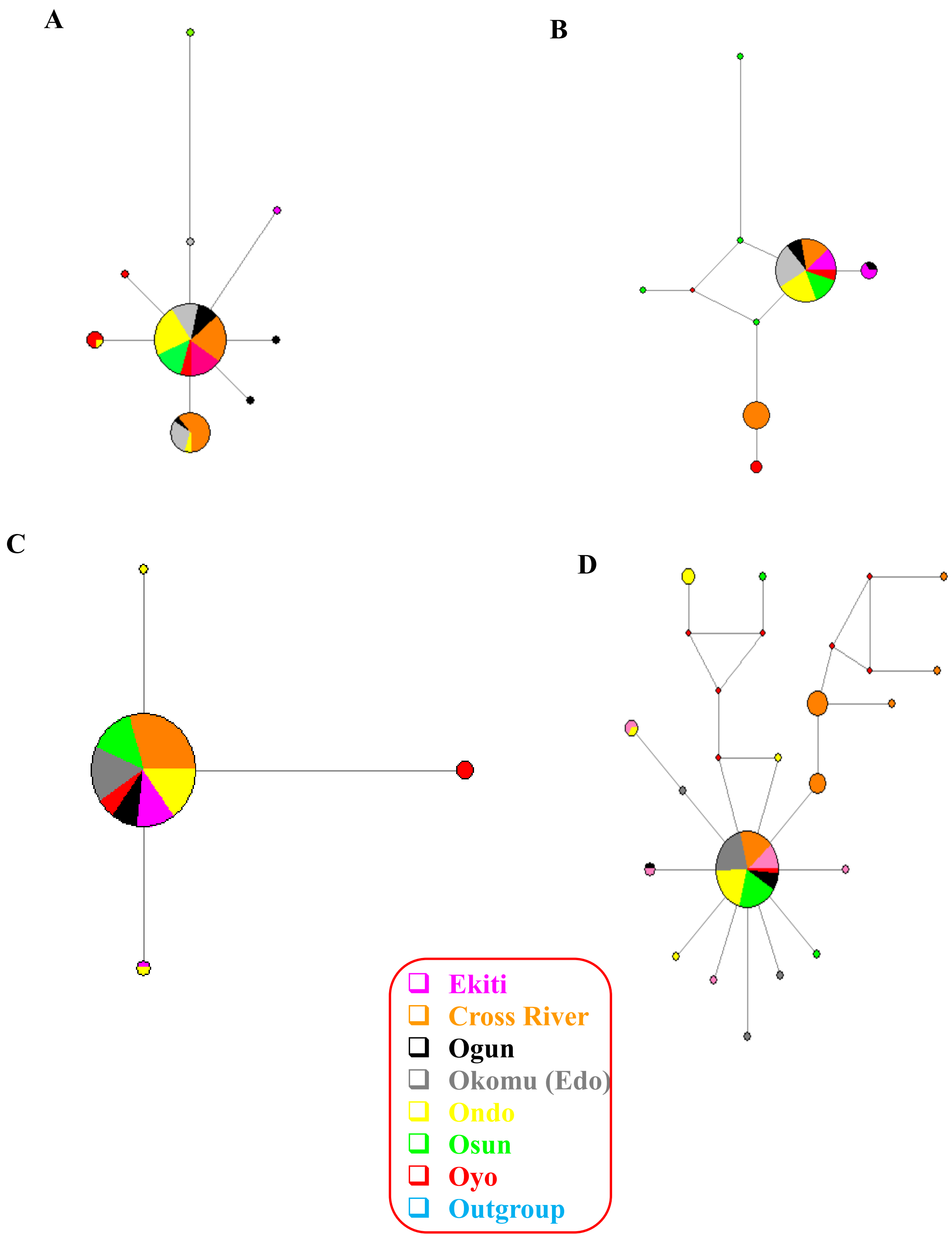

Figure S6. The haplotype networks for four independent mitochondrial regions of Nigerian grasscutter populations. (A) *CYTB* (B) 16S (C) 12S (D) *COI*. The haplotypes are coded according to the sampling locations. The size of each haplotype corresponds to the number of haplotypes.

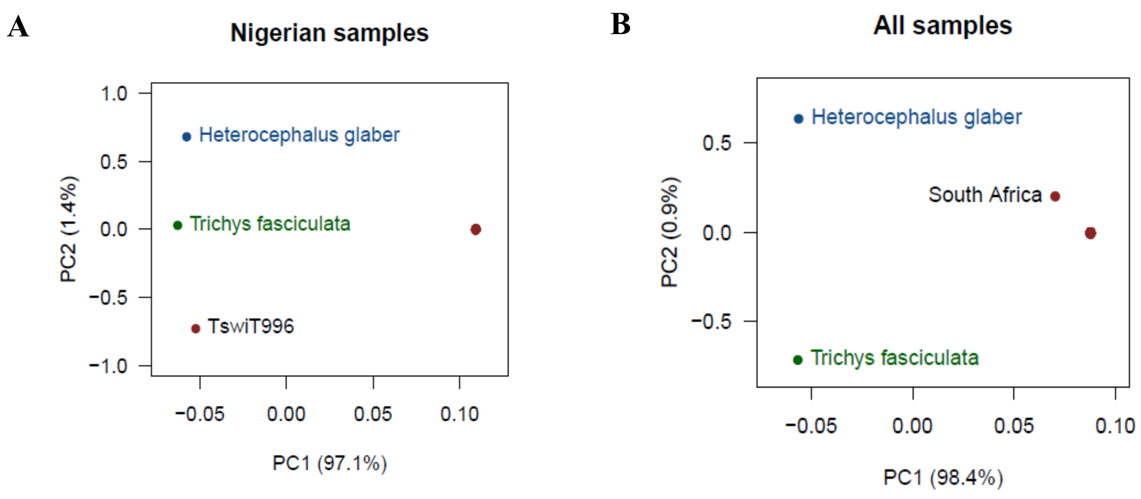

Figure S7. Principal component analyses based on the genetic distances of the 1,936 bp concatenated mitochondrial regions of grasscutter samples. The *Heterocephalus glaber* and *Trichis fasciculata* were used as outgroups. (A) PCA of Nigerian grasscutter samples identifies TswiT996 as an outlier. (B) PCA shows the uniformity of samples from investigated countries, after the removal of TswiT996. South African samples were found to be separated from other samples. Colors correspond to samples; Grasscutter samples – Dark red, *heterocephalus glaber* – Blue, *Trichys fasciculata* – Green.

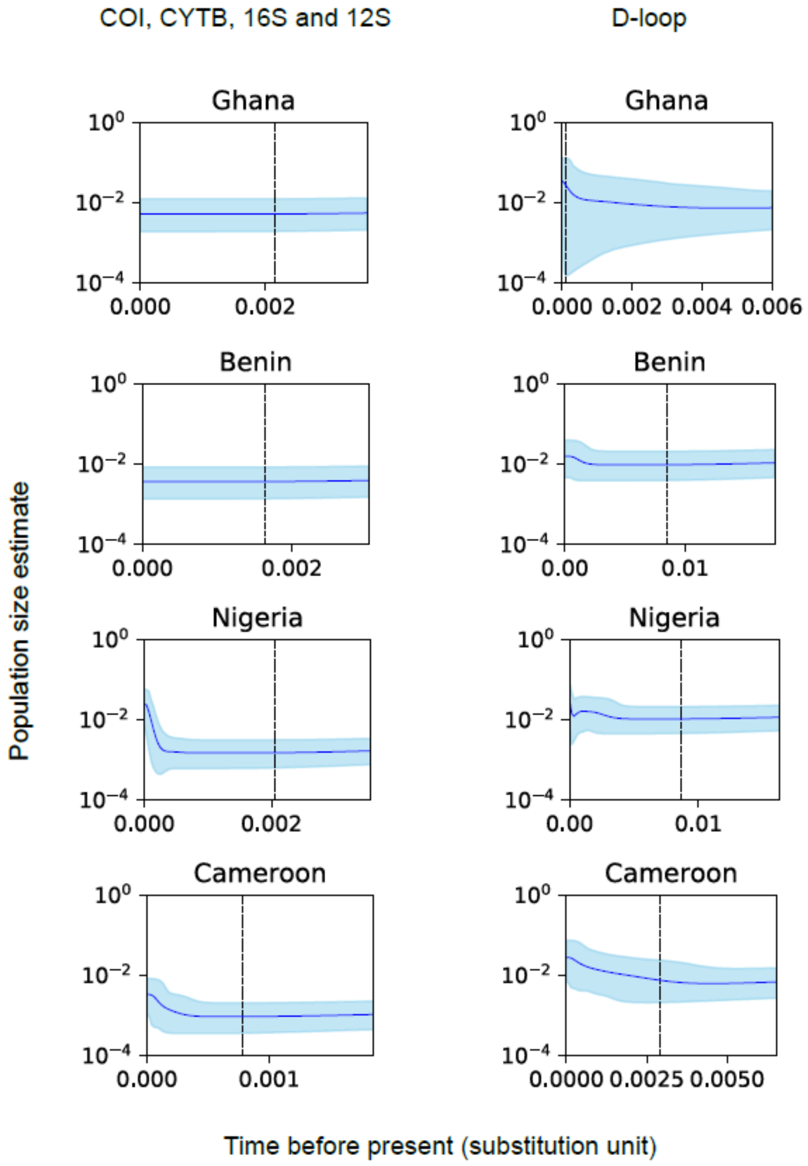

Figure S8. The skyline plots showing the demographic histories of the four countries with enough sample sizes. The plots at the left column were made from the concatenated COI, CYTB, 16S and 12S regions, while the plots at the right were computed from D-loop sequences.

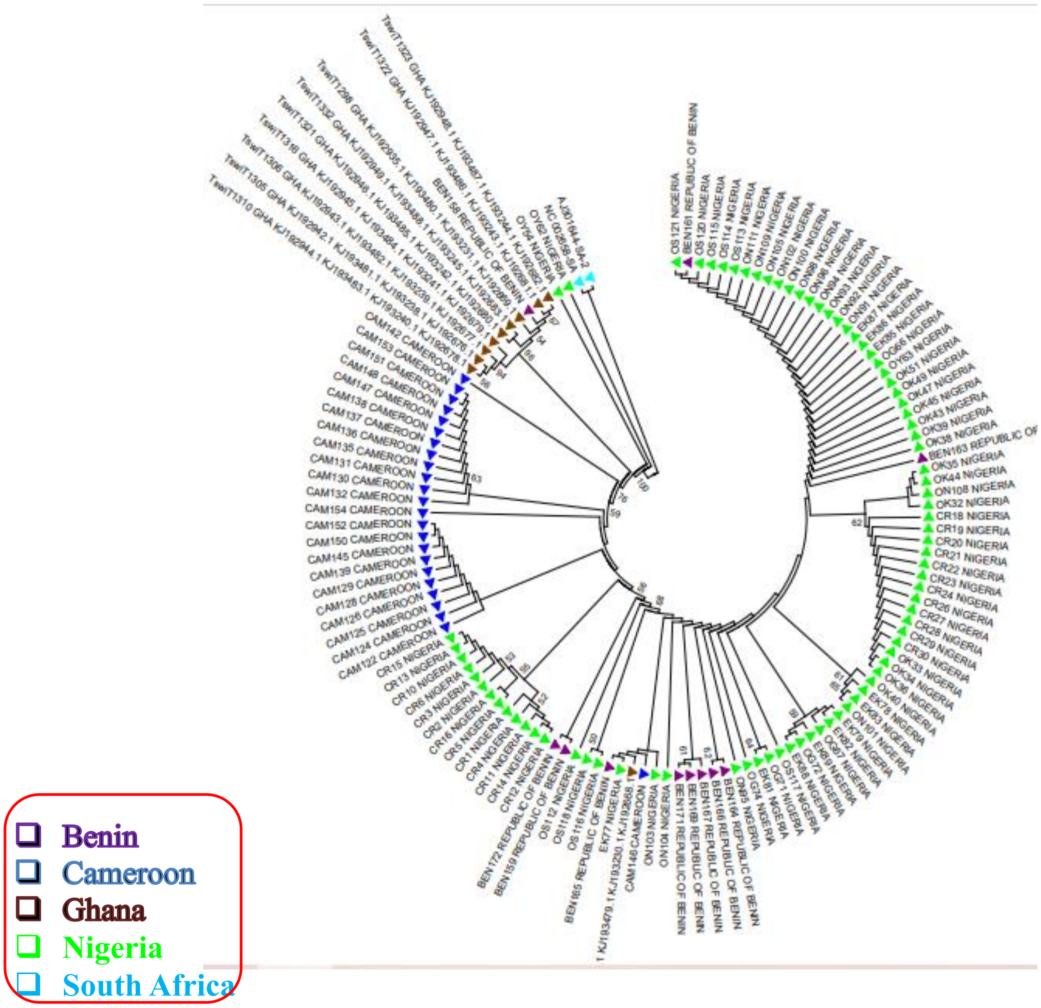

Figure S9. Maximum likelihood (ML) tree based on concatenated four mitochondrial regions of the grasscutter samples from Nigeria, Cameroon, Benin Republic and Ghana. Samples from South Africa were also included as outgroups. The samples are colored by the sampling location. The best tree from 1000 bootstraps is presented. Branches with bootstrap support of 50% are shown.

A

B

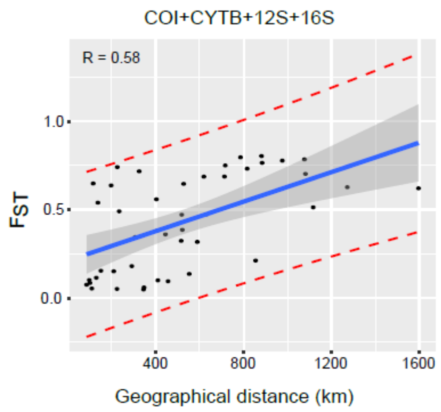

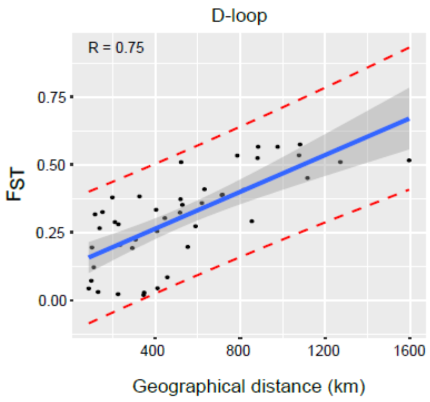

Figure S10. Relationship between geographical distance and genetic distance using concatenated COI, CYTB, 12S and 16S mitochondrial regions (A) and mitochondrial D-loop region (B). Each point represents a population pair. R represents the Pearson's coefficient of correlation between the geographical and genetic distances.

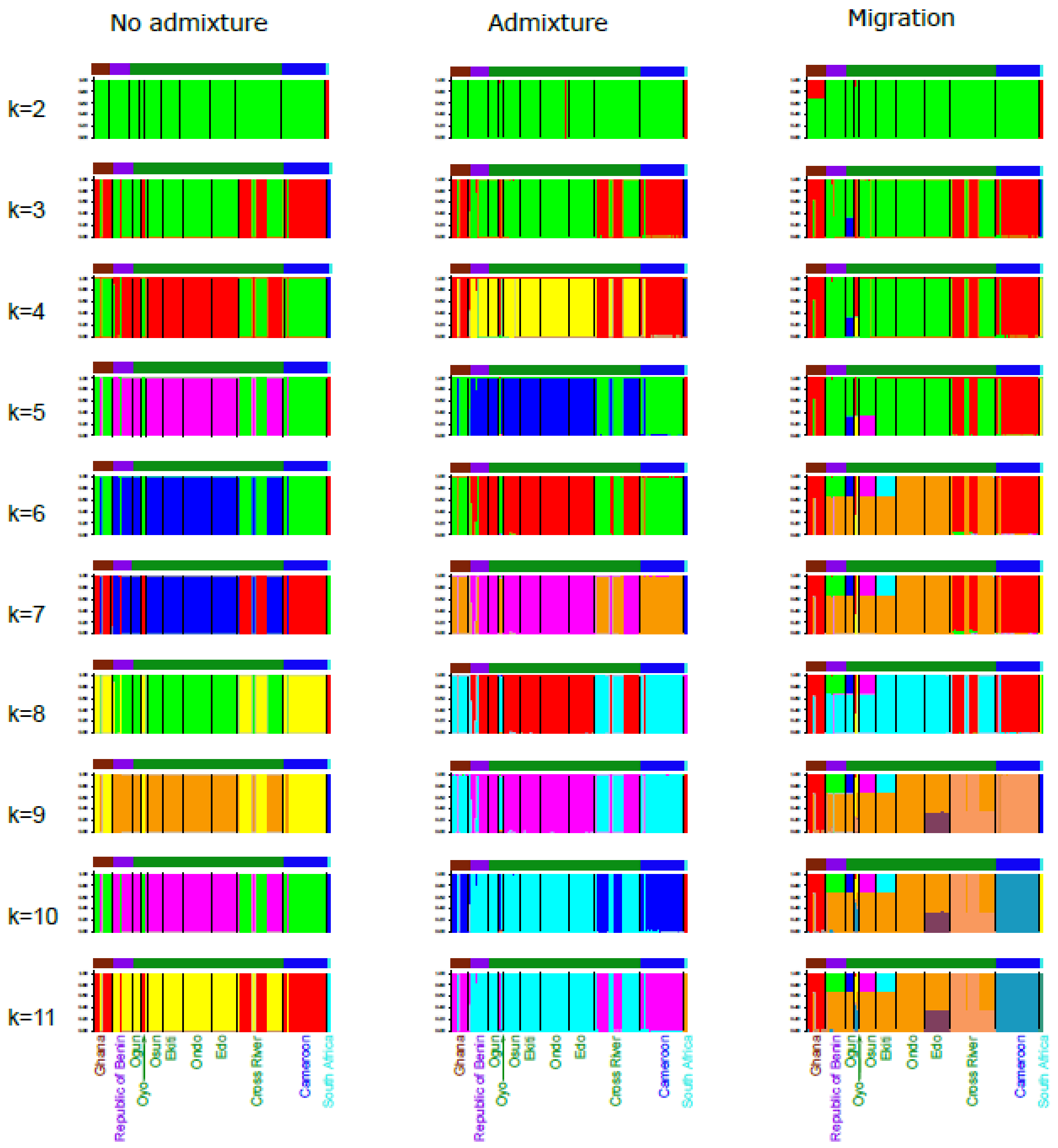

Figure S11. The genetic component analyses with STRUCTURE using the four merged mitochondrial regions (COI, CYTB, 12S and 16S). Three models with k values from 2 to 11 were investigated. The analyses were run in 10 replicates. The representative plot for each model and k value is presented. The left column shows the results for the model with no admixture, the middle column shows the results for the model with admixture, while the right column shows the results for the model involving migration.

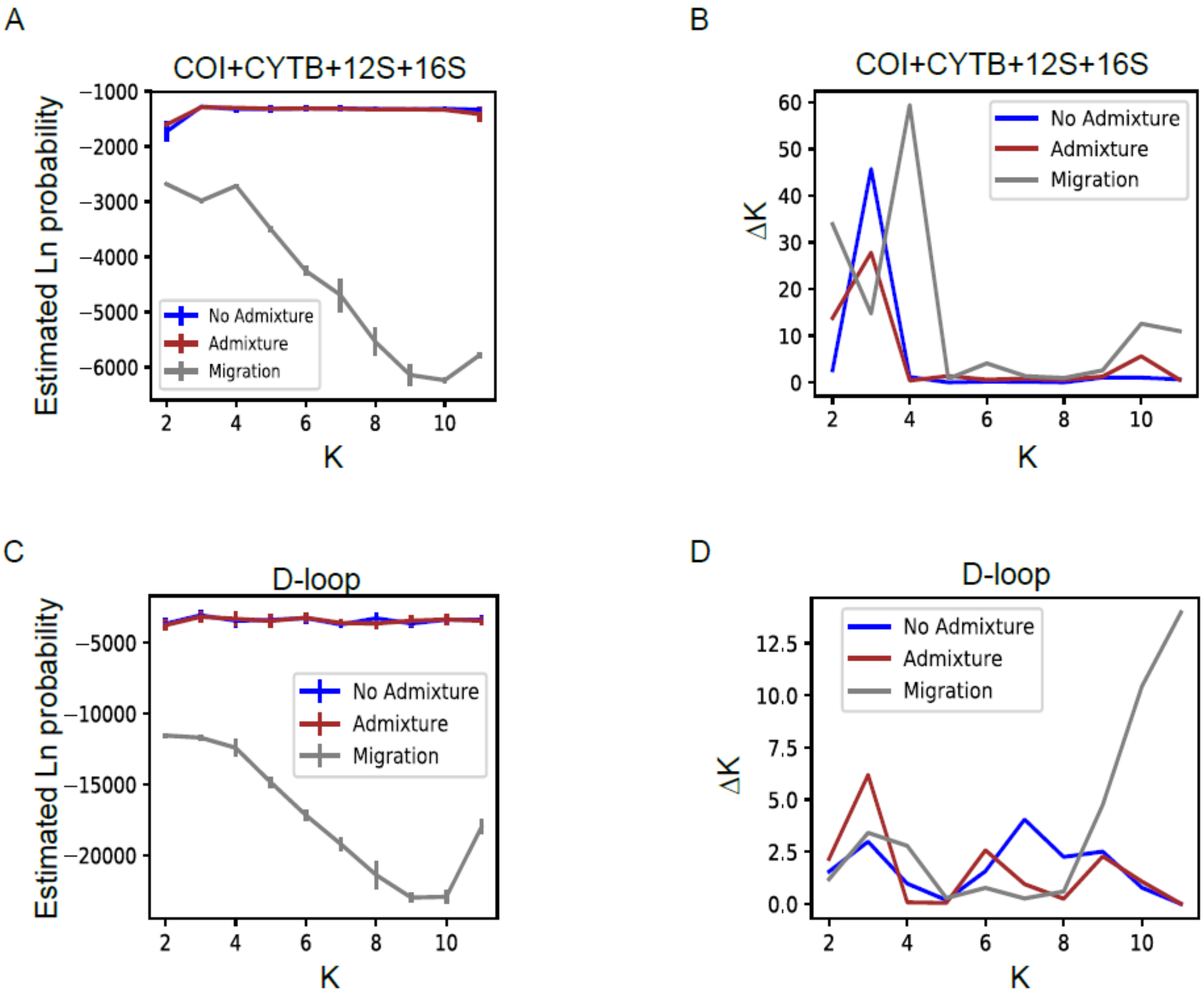

Figure 12: Investigation of various models and values of k for the analyses of genetic ancestry. Different K values (K=2 to K=11) were tested using three models in the STRUCTURE software. The models tested were models with no admixture, with admixture and model involving migration. The models were tested using the concatenated COI, CYTB, 12S and 16S mitochondrial regions and mitochondrial D-loop region. (A) STRUCTURE's model choice criterion for the concatenated COI, CYTB, 12S and 16S regions. (B) Evanno et al. (2005)'s Δk values for the concatenated COI, CYTB, 12S and 16S regions. (C) STRUCTURE's model choice criterion for the D-loop. (D) Evanno et al. (2005)'s Δk values for the D-loop. $\Delta K = m(|L(K+1) - 2\,L(K) + L(K-1)|)/s[L(K)]$; where L(K) is the data probability estimated from STRUCTURE for K (Evanno et al., 2005).

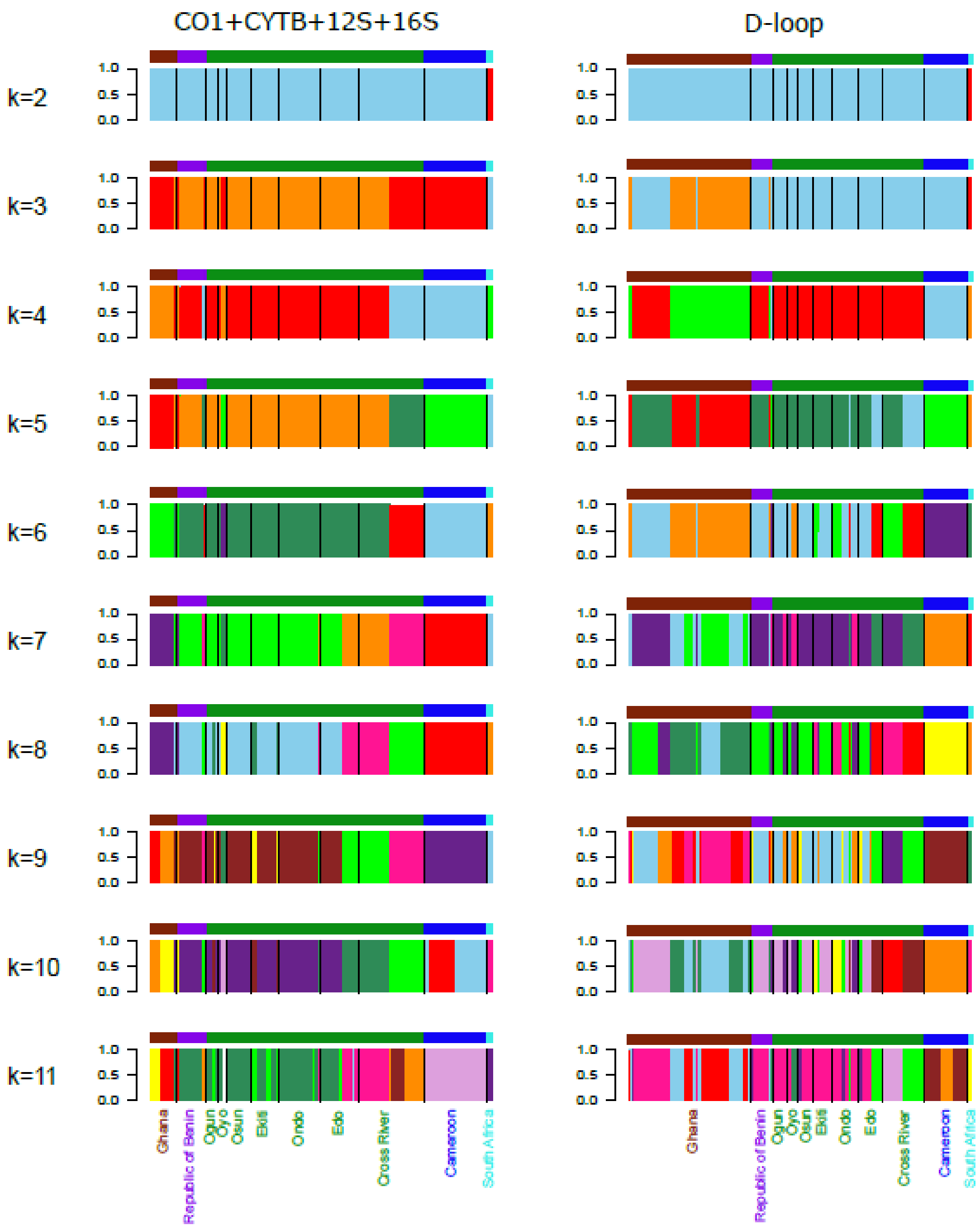

Figure S13. Genetic component analyses with DAPC. The results are presented for 2 to 11 clusters. The results of the four concatenated regions (COI, CYTB, 12S and 16S) are presented in the left column while the results from the D-loop are presented in the right column. In both cases, the first 30 PCs of the transformed data were used.

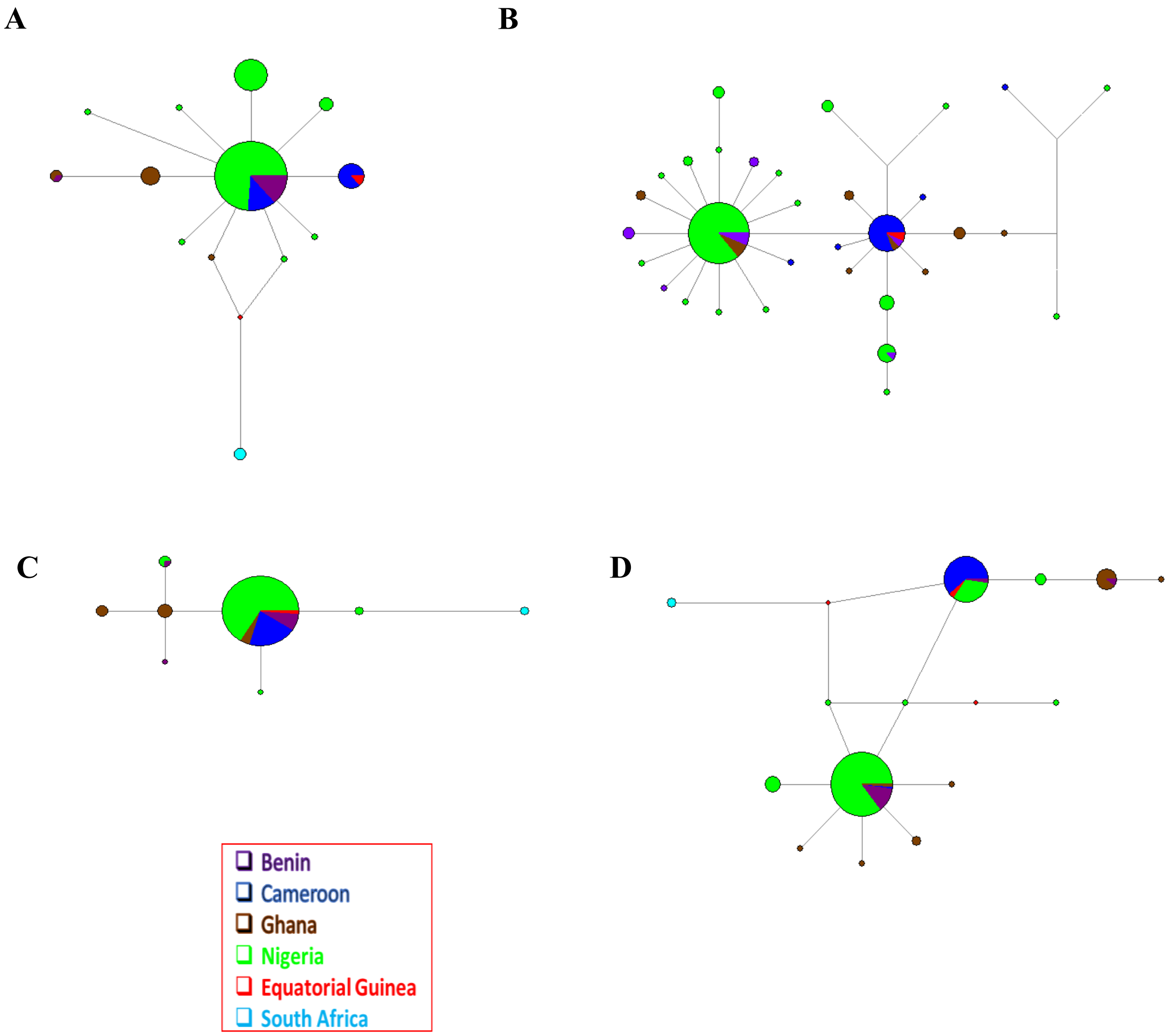

Figure S14. The haplotype networks for mitochondrial regions of African Guinea forest grasscutter populations including Benin, Cameroon, Ghana, Nigeria and Equatorial Guinea. (A) *CYTB* (B) *COI* (C) 12S haplotype network (D) 16S. Samples from South Africa were also included as the outgroup. The haplotypes are color-coded according to the sampling locations. The size of each haplotype corresponds to the number of samples with the haplotype.

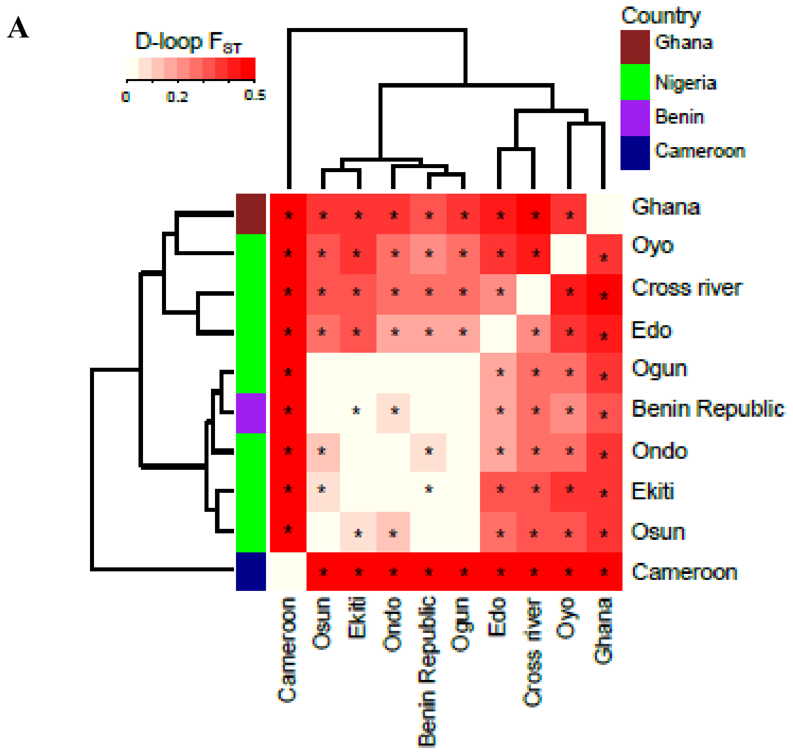

Figure S15. Population structures in various grasscutter populations and subpopulations using mitochondrial D-loop region. The heatmap shows the F_{ST} values between Nigerian subpopulations and populations from the other countries. The asterisks are significant at p-value of 0.05.

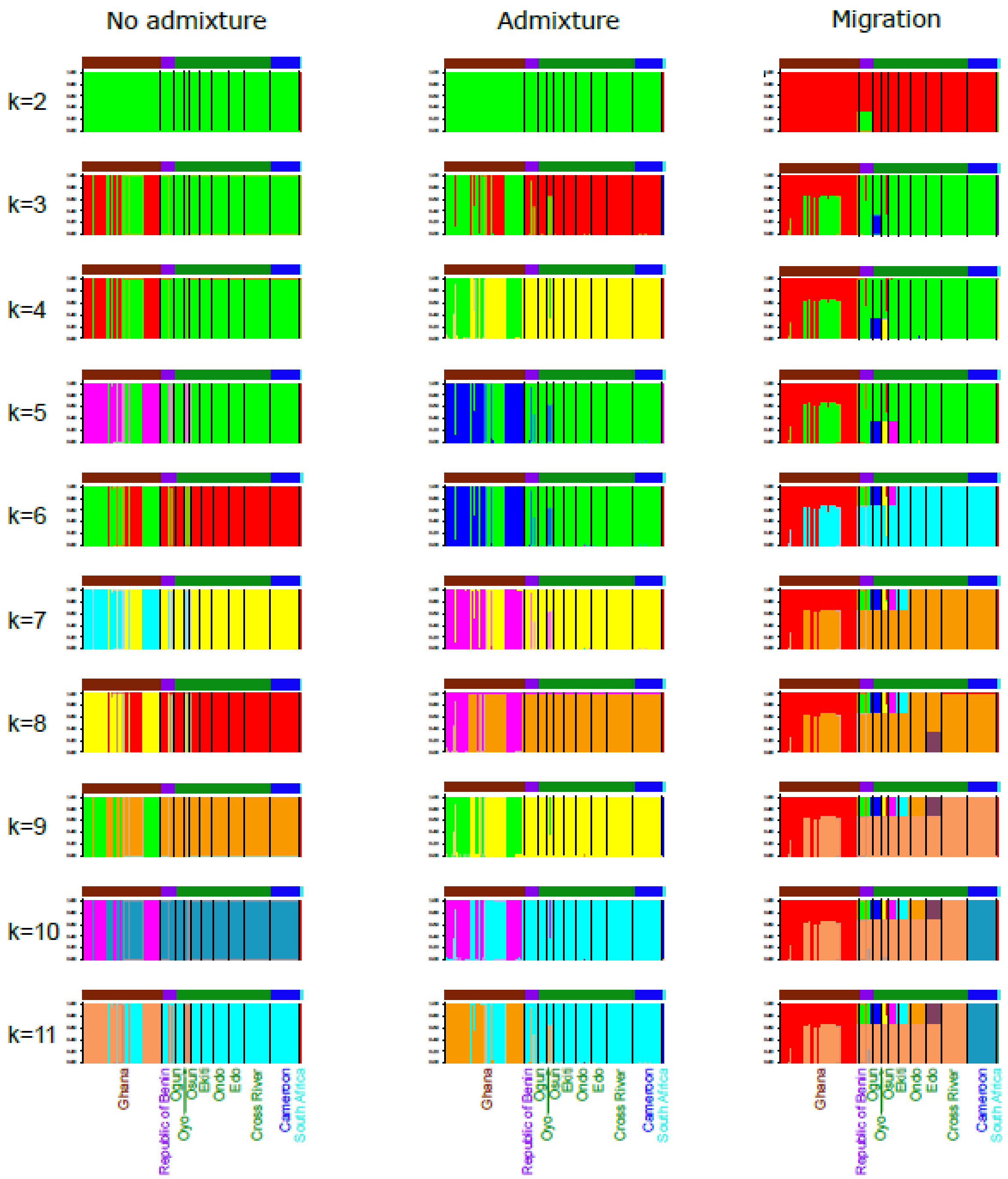

Figure S16. The genetic component analyses with STRUCTURE using the D-loop region. Three models with k values from 2 to 11 were investigated. The analyses were run in 10 replicates. The representative plot for each model and k value is presented. The left column shows the results for the model with no admixture, the middle column shows the results for the model with admixture, while the right column shows the results for the model involving migration.

2.2 Supplementary Tables

Table S2: Details of the single primer pairs used to amplify the five mitochondrial DNA fragments

Gene/Region	Primer pairs	Source	Annealing T° (C)	Product size (bp)
D-loop	CRmtDF 5’-CCAACTCCCAAAGCTGATGT-3’<br>CRmtDR 5’-GGCACCAACATCATCACAAA-3’	Adenyo et al. (2013)	55˚C	501
CYTB	GVL14724 5’GATATGAAAAACCATCGTTG3’<br>H15149 5’CTCAGAATGATATTTGTCCTCA3’	Gaubert *et al.* (2014)<br>Irwin *et al.* (1991)	50˚C	402
COI	bush-COIF 5’CACAAACCACAAAGAYATYGG3’<br>bush-COIR 5’TCAGGGTGTCCAAARAAYCA3’	Gaubert *et al.* (2014)	50˚C	658
16S	bush-16SF 5’CGCCTGTTTACCAAAAACATC3’<br>bush-16SR 5’AATCGTTGAACAAACGAACC3’	Gaubert *et al.* (2014)	52˚C	510–527
12S	bush-12SF 5’GGGATTAGATACCCCACTATGC3’<br>bush-12SR 5’GTGACGGGCGGTGTGT3’	Gaubert *et al.* (2014)	52˚C	384–430

Table S3: AMOVA results for the 2,437 bp of the five concatenated mitochondrial regions of 79 individuals sampled from seven Nigerian wild grasscutter populations.

Source of Variation	Degree of freedom	Sum of squares	Variance components	Percentage of variation
**Among populations**	6	109.274	1.42612 Va	32.31
**Within populations**	72	215.157	2.98829 Vb	67.69
**Total**	78	324.43	4.41441	

Significance tests (1023 permutations)

Va and FST : P(rand. value > obs. value) = 0.00000

P(rand. value = obs. value) = 0.00000

P-value = 0.00000+-0.00000

Table S4: F_{ST} values for pairwise comparisons of Nigerian wild grasscutter populations using 2,437 bp of the five concatenated mitochondrial regions**.**

	**Cross river**	**Ondo**	**Edo**	**Ekiti**	**Osun**	**Ogun**	**Oyo**
**Cross river**	*	0.000	0.007	0.003	0.007	0.021	0.000
**Ondo**	0.304	*	0.005	0.032	0.009	0.196	0.003
**Edo**	0.265	0.157	*	0.000	0.008	0.042	0.005
**Ekiti**	0.323	0.069	0.255	*	0.000	0.723	0.005
**Osun**	0.316	0.122	0.232	0.101	*	0.232	0.011
**Ogun**	0.273	0.039	0.196	-0.036	0.038	*	0.017
**Oyo**	0.532	0.623	0.651	0.656	0.647	0.544	*

The values at the lower-left of the are the FST while the upper-right values are the p values.

Table S5: P values for the pairwise differentiation tests based on the haplotype frequencies of the five concatenated mitochondrial regions of the Nigerian wild grasscutter populations.

	**Cross river**	**Ondo**	**Edo**	**Ekiti**	**Osun**	**Ogun**	**Oyo**
**Cross river**	*						
**Ondo**	0.000	*					
**Edo**	0.006	0.004	*				
**Ekiti**	0.000	0.040	0.011	*			
**Osun**	0.000	0.043	0.003	0.173	*		
**Ogun**	0.002	0.193	0.128	0.795	0.133	*	
**Oyo**	0.014	0.356	0.107	0.636	0.256	1.000	*

The test was conducted in Arlequin using 1,000,000 steps of Markov chain (Raymond and Rousset 1995)

Table S6: Haplotype sharing based on 1,936 bp concatenated four mitochondrial regions of Grasscutter samples from Nigeria, Ghana, Benin, Cameroon, and South Africa

Haplotype	HT Name	Frequency	Nigeria	Ghana	Benin	Cameroon	South Africa
HT1	NC_002658-SA	2	0	0	0	0	2
HT2	CR1_NIGERIA	5	5	0	0	0	0
HT3	CR2_NIGERIA	7	7	0	0	0	0
HT4	CR5_NIGERIA	1	1	0	0	0	0
HT5	CR17_NIGERIA	1	1	0	0	0	0
HT6	CR18_NIGERIA	16	16	0	0	0	0
HT7	OK32_NIGERIA	1	1	0	0	0	0
HT8	OK35_NIGERIA	1	1	0	0	0	0
HT9	OK38_NIGERIA	31	28	0	3	0	0
HT10	OK40_NIGERIA	1	1	0	0	0	0
HT11	OY54_NIGERIA	1	1	0	0	0	0
HT12	OY62_NIGERIA	1	1	0	0	0	0
HT13	OG67_NIGERIA	4	4	0	0	0	0
HT14	OG71_NIGERIA	2	2	0	0	0	0
HT15	OG72_NIGERIA	1	1	0	0	0	0
HT16	OG74_NIGERIA	1	1	0	0	0	0
HT17	EK77_NIGERIA	1	1	0	0	0	0
HT18	EK78_NIGERIA	3	3	0	0	0	0
HT19	EK88_NIGERIA	1	1	0	0	0	0
HT20	ON95_NIGERIA	1	1	0	0	0	0
HT21	ON103_NIGERIA	1	1	0	0	0	0
HT22	ON104_NIGERIA	1	1	0	0	0	0
HT23	ON108_NIGERIA	1	1	0	0	0	0
HT24	OS112_NIGERIA	1	1	0	0	0	0
HT25	OS116_NIGERIA	1	1	0	0	0	0
HT26	OS117_NIGERIA	1	1	0	0	0	0
HT27	OS118_NIGERIA	1	1	0	0	0	0
HT28	CAM122_CAMEROON	11	0	0	0	11	0
HT29	CAM130_CAMEROON	10	0	0	0	10	0
HT30	CAM132_CAMEROON	1	0	0	0	1	0

HT31	CAM142_CAMEROON	1	0	0	0	1	0
HT32	CAM146_CAMEROON	1	0	0	0	1	0
HT33	BEN158_BENIN	3	0	2	1	0	0
HT34	BEN159_BENIN	1	0	0	1	0	0
HT35	BEN164_BENIN	2	0	0	2	0	0
HT36	BEN167_BENIN	3	0	0	3	0	0
HT37	BEN172_BENIN	1	0	0	1	0	0
HT38	TswiT1310_GHA	1	0	1	0	0	0
HT39	TswiT1316_GHA	2	0	2	0	0	0
HT40	TswiT1297_GHA	1	0	1	0	0	0
HT41	TswiT996_GHA	1	0	1	0	0	0
HT42	TswiT1298_GHA	1	0	1	0	0	0
HT43	TswiT1305_GHA	1	0	1	0	0	0
HT44	TswiT1321_GHA	1	0	1	0	0	0
HT45	TswiT1332_GHA	1	0	1	0	0	0

Table S7: Exact test of differentiation based on haplotype frequencies for the concatenated four mitochondrial regions

	Nigeria	Cross river	Cameroon	Ondo	Edo	Ekiti	Benin	Ghana	Osun	Ogun	Oyo	South Africa
**Nigeria**												
**Cross river**	1.05E-02											
**Cameroon**	0.00E+00	0.00E+00										
**Ondo**	3.74E-01	0.00E+00	0.00E+00									
**Edo**	9.00E-01	2.00E-05	0.00E+00	4.39E-02								
**Ekiti**	2.21E-01	0.00E+00	0.00E+00	2.76E-02	9.63E-03							
**Benin**	1.63E-03	0.00E+00	0.00E+00	8.32E-03	8.87E-03	3.94E-02						
**Ghana**	0.00E+00	0.00E+00	0.00E+00	1.00E-04	3.60E-04	5.68E-03	5.15E-03					
**Osun**	3.75E-01	0.00E+00	0.00E+00	4.07E-01	1.13E-01	1.17E-01	1.19E-01	8.63E-03				
**Ogun**	1.92E-01	8.00E-05	5.10E-04	8.06E-02	6.50E-02	6.56E-01	1.88E-01	2.64E-01	2.39E-01			
**Oyo**	2.76E-01	1.03E-03	6.17E-03	2.93E-01	2.40E-01	3.63E-01	3.65E-01	3.57E-01	5.03E-01	1.00E+00		
**South Africa**	4.35E-02	2.99E-03	1.29E-02	7.10E-02	3.41E-02	6.40E-02	6.47E-02	2.73E-01	1.26E-01	5.24E-01	4.00E-01	

The test was conducted in Arlequin using 1,000,000 steps of Markov chain (Raymond and Rousset 1995)

Table S8: Geographical structure assessed using analysis of molecular variance (AMOVA). AMOVA subdivides genetic diversity into hierarchical components and estimates the parameter $F_{ST,}$ which assesses the relative divergence between populations. The statistical significance of variance components and F_{ST} indices were assessed by bootstrapping using 1,000 replicates. Analyses were carried out sequentially for all samples (132-1936 bp of four mtDNA regions of Grasscutters from Nigeria, Cameroon, Benin, Ghana, South Africa)

Hierarchical clusters	Hierarchy	Variance components	% of variation	F	*P*-value
1. Nigerian grasscutter populations (overall)[1]	1	Within populations	83.59	-	-
		Among populations	16.41	0.16412	0.00978
2. CRS vs. remaining populations[2]	2	Within populations	75.78	0.24215	0.01271
		Within populations within groups	4.36	0.05438	0.00000
		Among groups	19.86	0.19857	0.30010
3. OK vs. remaining populations[2]	2	Within populations	89.39	0.10613	0.00684
		Within populations within groups	21.19	0.19166	0.03715
		Among groups	-10.58	-0.10582	0.71065
4. EK vs. remaining populations[2]	2	Within populations	88.70	0.11300	0.00880
		Within populations within groups	19.82	0.18264	0.02346
		Among groups	-8.52	-0.08519	0.44086
5. OG vs. remaining populations[2]	2	Within populations	93.43	0.06570	0.00978
		Within populations within groups	20.24	0.17807	0.02053
		Among groups	-13.67	-0.13671	1.00000
6. ON vs. remaining populations[2]	2	Within populations	88.86	0.11141	0.01369
		Within populations within groups	21.35	0.19372	0.04692
		Among groups	-10.21	-0.10210	0.57674

7. OS vs. remaining populations[2]	2	Within populations	91.46	-0.12342	0.01075
		Within populations within groups	20.88	0.18590	0.02346
		Among groups	-12.34	0.08543	0.83969
8. OY vs. remaining populations[2]	2	Within populations	56.14	0.43862	0.00978
		Within populations within groups	7.96	0.12414	0.00000
		Among groups	35.91	0.35905	0.14663
9. CRS + OK vs. remaining populations[2]	2	Within populations	81.84	0.18163	0.01760
		Within populations within groups	12.67	0.13410	0.00000
		Among groups	5.49	0.05490	0.20821
10. CRS vs OK vs. remaining populations[2]	3	Within populations	72.99	0.27005	0.00978
		Among populations within groups	-3.74	-0.05405	0.00000
		Among groups	30.75	0.30748	0.04008
11. CRS + OK vs OY vs. remaining populations[2]	3	Within populations	79.21	0.20787	0.01173
		Among populations within groups	5.32	0.06293	0.00098
		Among groups	15.47	0.15467	0.08113
12.Grasscutter populations (overall)[3]	1	Within populations	74.97	-	-
		Among populations	25.03	0.25027	0.00000
13. NIG vs. remaining populations[3]	2	Within populations	76.08	0.23922	0.00000
		Within populations within groups	34.18	0.30997	0.00000
		Among groups	-10.25	-0.10254	0.73607
14. CAM vs. remaining populations[3]	2	Within populations	76.87	0.23128	0.00000
		Within populations within groups	28.93	0.27346	0.00000
		Among groups	-5.80	-0.05805	0.50831

15. BEN vs. remaining populations[3]	2	Within populations	91.22	0.08784	0.00000
		Within populations within groups	39.30	0.30112	0.00000
		Among groups	-30.52	-0.30517	1.00000
16. GHA vs. remaining populations[3]	2	Within populations	59.08	0.40919	0.00000
		Within populations within groups	11.06	0.15771	0.00000
		Among groups	29.86	0.29856	0.25513
17. NIG + CAM vs GHA + BEN	2	Within populations	76.62	0.23381	0.00000
		Among populations within groups	28.02	0.26776	0.00000
		Among groups	-4.64	-0.04636	0.68524
18. GHA vs BEN vs NIG + CAM	3	Within populations	72.41	0.27588	0.00000
		Among populations within groups	19.94	0.21593	0.04203
		Among groups	7.65	0.07646	0.33920
19. GHA vs BEN + NIG vs CAM	3	Within populations	70.13	0.29875	0.00000
		Among populations within groups	-1.70	-0.02491	0.30792
		Among groups	31.58	0.31579	0.17302
20. GHA + BEN vs NIG vs CAM	3	Within populations	75.50	0.24500	0.00000
		Within populations within groups	50.74	0.40194	0.00000
		Among groups	-26.24	-0.26242	0.84164
21. SA vs. remaining populations[6]	2	Within populations	1.39	0.98606	0.00000
		Within populations within groups	0.38	0.21500	0.00000
		Among groups	98.22	0.98225	0.08016
22. Grasscutter populations (overall)[5]	1	Within populations	27.18	-	-
		Among populations	72.82	0.72819	0.00000

[1]Overall is Nigerian Grasscutter populations which were as follows (number of samples in parentheses): CR (Cross River; n=26), EK (Ekiti; n=11), OG (Ogun; n=5), OK (Okomu-Edo; n=14), ON (Ondo; n=16), OS (Osun; n=9), OY (Oyo; n=3)

[2]Remaining populations listed above except the mentioned population.

[3]Overall is west Africa Grasscutter populations which were as follows (number of samples in parentheses): NIG (Nigeria; n=84), CAM (Cameroon; n=24), BEN (Benin; n=11), and GHA (Ghana; n=11)

[4]Remaining populations include all the four populations within west Africa listed above except the mentioned population.

[5]Overall is west Africa Grasscutter populations which were as follows (number of samples in parentheses): NIG (Nigeria; n=84), CAM (Cameroon; n=24), BEN (Benin; n=11), GHA (Ghana; n=11), and SA (South Africa; n=2)

[6]Remaining populations include all the five populations listed above except the mentioned population.

Table S9: D-Loop haplotype sharing among grasscutter populations from Ghana, Benin, Nigeria, and Cameroon

S/No	HT Name	Frequency	Ghana	Benin	Nigeria	Cameroon
1	AB675407_G6-23	**2**	1	1	0	0
2	BEN159	**1**	0	1	0	0
3	AB675395_GC1-11-1	**45**	12	10	22	1
4	BEN162	**1**	0	1	0	0
5	AB675397_C5-13	**10**	1	1	8	0
6	OK39	**6**	0	1	5	0
7	CAM122	**8**	0	0	0	8
8	CAM124	**2**	0	0	0	2
9	CAM126	**1**	0	0	0	1
10	CAM127	**7**	0	0	0	7
11	AB675385_GFC1-1-1	**9**	9	0	0	0
12	AB675386_GF-2-1	**6**	6	0	0	0
13	AB675387_FC-3-1	**14**	14	0	0	0
14	AB675388_F1-4-1	**3**	3	0	0	0
15	AB675389_GFC2-5-1	**5**	5	0	0	0
16	AB6675390_F2-6	**1**	1	0	0	0
17	AB675391_F3-7	**1**	1	0	0	0
18	AB675392_C1-8	**1**	1	0	0	0
19	AB675393_C2-9	**1**	1	0	0	0
20	AB675394_C3-10	**1**	1	0	0	0
21	AB675396_C4-12-1	**9**	9	0	0	0
22	AB675398_C6-14	**1**	1	0	0	0
23	AB675399_GC2-15-1	**6**	6	0	0	0
24	AB675400_GC3-16-1	**3**	3	0	0	0
25	AB675401_C7-17	**1**	1	0	0	0
26	AB675402_G1-18	**1**	1	0	0	0
27	AB675403_G2-19	**1**	1	0	0	0
28	AB675404_G3-20	**1**	1	0	0	0

29	AB675405_G4-21	**1**	1	0	0	0
30	AB675406_G5-22	**1**	1	0	0	0
31	AB675408_G7-24	**1**	1	0	0	0
32	AB675409_G8-25	**1**	1	0	0	0
33	AB675410_G9-26	**1**	1	0	0	0
34	CAM131	**2**	0	0	0	2
35	CAM132	**2**	0	0	0	2
36	CAM136	**1**	0	0	0	1
37	CAM138	**1**	0	0	0	1
38	CAM142	**1**	0	0	0	1
39	CAM149	**2**	0	0	0	2
40	CAM154	**2**	0	0	0	2
41	CAM156	**1**	0	0	0	1
42	CR1	**5**	0	0	5	0
43	CR2	**8**	0	0	8	0
44	CR12	**1**	0	0	1	0
45	CR17	**23**	0	0	23	0
46	EK77	**1**	0	0	1	0
47	EK80	**10**	0	0	10	0
48	EK82	**2**	0	0	2	0
49	EK85	**1**	0	0	1	0
50	EK86	**1**	0	0	1	0
51	OG69	**5**	0	0	5	0
52	OG72	**1**	0	0	1	0
53	OK40	**1**	0	0	1	0
54	OK48	**1**	0	0	1	0
55	OK49	**1**	0	0	1	0
56	ONB105	**1**	0	0	1	0
57	ONB109	**2**	0	0	2	0
58	OS115	**1**	0	0	1	0
59	OS117	**1**	0	0	1	0
60	OY54	**3**	0	0	3	0

Table S10: Genetic diversity of Grasscutter populations based on mitochondrial D-loop

Population	N	nHT	Htdiv	πdiv	*D*	*Fs*	SSD	HRI	MNPD
Cross River	28	4	0.659 (0.059)	0.458 (0.262)	2.473	5.71	0.162	0.227*	4.124(2.116)
Ekiti	14	7	0.758 (0.116)	0.237 (0.166)	-0.908	-2.672*	0.017	0.073	1.659(1.037)
Ogun	10	5	0.756 (0.130)	0.258 (0.178)	-1.161	-0.528	0.024	0.08	2.067(1.261)
Okomu (Edo)	17	6	0.743 (0.089)	0.257 (0.165)	-0.478	-0.179	0.07	0.261*	2.309(1.329)
Ondo	18	9	0.837 (0.075)	0.214 (0.127)	-1.012	-1.431	0.034	0.092	3.641(1.934)
Osun	10	4	0.711 (0.118)	0.253 (0.196)	-1.136	-0.384	0.032	0.177	1.267(0.868)
Oyo	7	3	0.714 (0.127)	0.551 (0.335)	1.926	4.854	0.342*	0.776*	7.714(4.095)
Nigeria	104	27	0.912 (0.015)	0.009 (0.005)	-1.209*	-7.521*	0.008	0.027	4.688(2.315)
Benin	15	6	0.571 (0.149)	0.007 (0.004)	-1.856*	0.719	0.392*	0.127	3.543(1.908)
Cameroon	31	13	0.884 (0.038)	0.006 (0.004)	-0.656*	-3.688*	0.02	0.048	3.226(1.711)
Ghana	84	26	0.921 (0.013)	0.012 (0.007)	1.079	-4.874*	0.003	0.008	4.688(2.315)

N=Total number of samples analyzed; nHT=Number of haplotypes; HTdiv=Haplotype diversity, πdiv=Nucleotide diversity; *D*=Tajima's *D* test of selective neutrality; *Fs*=Fu's *Fs* test of selective neutrality; SSD=Sum of square deviation for mismatch distribution; HRI= Harpending's raggedness index for mismatch distribution; MNPD=Mean number of pairwise differences. The values in braces are the standard deviations. Asterisks indicate statistical significance at 5% level. Benin=Republic of Benin.

Table S11: Genetic diversity of Grasscutter population based on *CYTB*

Population	Size[a]	nHT[b]	HTdiv (SD)[c]	πdiv (SD)[d]	*D*[e]	*Fs*[f]	MNPD (SD)[g]
Cross River	30	2	0.515(0.027)	0.515(0.497)	1.621	1.700	0.515(0.446)
Ekiti	12	2	0.167(0.134)	0.167(0.201)	-1.451*	0.432	0.333(0.356)
Ogun	10	4	0.533(0.180)	0.200(0.196)	-1.562*	-1.964*	0.600(0.519)
Okomu (Edo)	17	3	0.581(0.068)	0.316(0.291)	0.172	0.109	0.632(0.520)
Ondo	19	3	0.205(0.119)	0.105(0.149)	-1.511*	-1.804*	0.211(0.267)
Osun	11	2	0.181(0.144)	0.182(0.100)	-2.231*	14.678	14.364(6.980)
Oyo	7	3	0.714(0.127)	0.429(0.391)	0.206	-0.237	0.857(0.682)
Nigeria	106	9	0.481(0.049)	0.024(0.015)	-2.834*	-0.033	2.019(1.146)
Benin	15	2	0.133(0.112)	0.133(0.173)	-1.491*	0.235	0.267(0.309)
Cameroon	29	2	0.512(0.031)	0.512(0.496)	1.595	1.668	0.512(0.445)
Ghana	10	3	0.511(0.164)	0.252(0.228)	-1.034	-0.046	0.756(0.604)
South Africa	3	1	-	-	-	-	-
Equatorial Guinea	2	1	-	-	-	-	-

[a]Total number of samples in each region, [b]Number of haplotypes, [c]Haplotype diversity (standard deviation), [d]Nucleotide diversity (standard deviation), [e]Tajima's *D* test of selective neutrality, [f]Fu's *Fs* test of selective neutrality, [g]Mean number of pairwise differences (standard deviation). Asterisks indicate statistical significance at 5% level.

Table S12: Geographical structure assessed using analysis of molecular variance (AMOVA). AMOVA subdivides genetic diversity into hierarchical components and estimates the parameter $F_{ST,}$ which assesses the relative divergence between populations. The statistical significance of variance components and F_{ST} indices were assessed by bootstrapping using 1000 replicates. Analyses were carried out sequentially for all samples (D-loop)

Hierarchical clusters	Hierarchy	Variance components	% of variation	F	*P*-value
1. Nigerian populations (overall)[1]	1	Within populations	86.14	-	-
		Among populations	13.86	0.13861	0.00000
2. CRS+EDO vs. remaining populations[3]	2	Within populations	69.71	0.30290	0.00000
		Among populations within groups	15.65	0.18336	0.00000
		Among groups	14.64	0.14638	0.04790
3. EDO vs. remaining populations[3]	2	Within populations	77.12	0.22881	0.00000
		Among populations within groups	28.87	0.27240	0.00000
		Among groups	-5.99	-0.05990	0.42131
4. CRS vs. remaining populations[3]	2	Within populations	67.37	0.32632	0.00000
		Among populations within groups	15.26	0.18464	0.00000
		Among groups	17.38	0.17376	0.29130
5. OYO vs. remaining populations[3]	2	Within populations	56.16	0.43840	0.00000
		Among populations within groups	15.26	0.21370	0.00000
		Among groups	28.58	0.28577	0.13978

6. EKITI vs. remaining populations[3]	2	Within populations	79.79	0.20213	0.00000
		Among populations within groups	30.80	0.27852	0.00000
		Among groups	-10.59	-0.10589	0.70381
7. OGUN vs. remaining populations[3]	2	Within populations	84.16	0.15845	0.00000
		Among populations within groups	32.93	0.28125	0.00000
		Among groups	-17.09	-0.17086	1.00000
8. ONDO vs. remaining populations[3]	2	Within populations	79.68	0.20316	0.00000
		Among populations within groups	31.73	0.28479	0.00000
		Among groups	-11.41	-0.11415	0.86608
9. OSUN vs. remaining populations[3]	2	Within populations	79.70	0.20302	0.00000
		Among populations within groups	29.78	0.27204	0.00000
		Among groups	-9.48	-0.09480	0.57380
10. West African populations (overall)[1]	1	Within populations	58.98	-	-
		Among populations	41.02	0.41020	0.00000
11. GHA + BEN vs. NIG + CAM	2	Within populations	58.91	0.41094	0.00000
		Among populations within groups	40.59	0.40798	0.00000
		Among groups	0.50	0.00500	0.65200
12. GHA vs. remaining populations[2]	2	Within populations	56.80	0.43201	0.00000
		Within populations within groups	30.62	0.35030	0.00000
		Among groups	12.58	0.12576	0.50147

13. BEN vs. remaining populations[2]	2	Within populations	74.33	0.25671	0.00000
		Within populations within groups	57.56	0.43644	0.00000
		Among groups	-31.89	-0.31893	1.00000
14. NIG vs. remaining populations[2]	2	Within populations	61.87	0.38131	0.00000
		Within populations within groups	58.32	0.48526	0.00000
		Among groups	-20.19	-0.20194	0.75269
15. CAM vs. remaining populations[2]	2	Within populations	52.14	0.47861	0.00000
		Within populations within groups	29.95	0.36481	0.00000
		Among groups	17.92	0.17916	0.25513
16. GHA vs. BEN+NIG vs. CAM	3	Within populations	56.87	0.43133	0.00000
		Among populations within groups	2.14	0.03619	0.04203
		Among groups	41.00	0.40998	0.16716
17. GHA+BEN vs. NIG vs. CAM	3	Within populations	58.66	0.41338	0.00000
		Among populations within groups	33.69	0.36477	0.00000
		Among groups	7.65	0.07653	0.33040
18. GHA vs. BEN vs. NIG + CAM	3	Within populations	59.40	0.40596	0.00000
		Among populations within groups	44.57	0.42869	0.00000
		Among groups	-3.98	-0.03978	0.84262

[1]Overall is Grass cutter populations which were as follows (number of samples in parentheses): Ghana (GHA; n=84), Benin (BEN; n=15), Nigeria (NIG; n=104) and Cameroon (CAM; n=31).

[2]remaining populations include all the four populations within west Africa listed above except the mentioned population.

[3]remaining populations include all the seven populations within Nigeria listed above except the mentioned population.

Table S13: Geographical structure assessed using analysis of molecular variance (AMOVA). AMOVA subdivides genetic diversity into hierarchical components and estimates the parameter F_{ST}, which assesses the relative divergence between populations. The statistical significance of variance components and F_{ST} indices were assessed by bootstrapping using 1000 replicates. Analyses were carried out sequentially for all samples (*CYTB*)

**Hierarchical clusters**	**Hierarchy**	**Variance components**	**% of variation**	**F**	***P*-value**
1. Nigerian populations (overall)[1]	1	Within populations	92.17	-	-
		Among populations	7.83	0.07833	0.00000
2. CR+OG+EDO+ON vs. remaining populations[3]	2	Within populations	89.65	0.10351	0.00000
		Within populations within regions	4.98	0.05258	0.00489
		Among regions	5.38	0.05376	0.00196
3. OYO+ON vs. remaining populations[3]	2	Within populations	92.84	0.07161	0.00000
		Within populations within regions.	8.48	0.08373	0.00000
		Among regions	-1.32	-0.01322	0.67840
4. EDO+CR vs. remaining populations[3]	2	Within populations	89.82	0.10181	0.00000
		Within populations within regions	3.84	0.04102	0.03226
		Among regions	6.34	0.06339	0.04790
5. OYO vs. remaining populations[2]	2	Within populations	91.46	0.08544	0.00000
		Within populations within groups	7.64	0.07707	0.00000
		Among groups	0.91	0.00907	0.31281
6. OSUN vs. remaining populations[2]	2	Within populations	75.15	0.24852	0.00000
			1.01	0.01330	0.00000

		Within populations within groups	23.84	0.23840	0.00000
		Among groups			
7. EDO vs. remaining populations[2]	2	Within populations	95.87	0.04128	0.00000
		Within populations within groups	10.10	0.09527	0.00000
		Among groups	-5.97	-0.05967	0.99707
8. EKITI vs. remaining populations[2]	2	Within populations	96.33	0.03670	0.00000
		Within populations within groups	9.64	0.09096	0.00000
		Among groups	-5.97	-0.05969	0.99609
9. CR vs. remaining populations[2]	2	Within populations	92.14	0.07862	0.00000
		Within populations within groups	7.80	0.07805	0.00000
		Among groups	0.06	0.00062	0.39883
10. OG vs. remaining populations[2]	2	Within populations	98.95	0.01054	0.00000
		Within populations within groups	10.33	0.09453	0.00000
		Among groups	-9.28	-0.09276	1.00000
11. ON vs. remaining populations[2]	2	Within populations	96.23	0.03770	0.00000
		Within populations within groups	10.62	0.09940	0.00000
		Among groups	-6.85	-0.06851	1.00000
12.CR+EDO vs. OY+OG vs. OS+ON+EK	3	Within populations	91.22	0.08776	0.00000
		Within populations within regions	4.65	0.04849	0.00000
		Among regions	4.13	0.04127	0.10753

13.OY+OG+ON+EKITI+OS vs. EDO vs. CR	3	Within populations	92.10	0.07896	0.00000
		Within populations within regions	7.66	0.07680	0.00098
		Among regions	0.23	0.00234	0.39980
14.OY+OS+OG vs. CR+EDO vs. EK+ON	3	Within populations	91.09	0.08912	0.00000
		Within populations within regions	3.46	0.03655	0.99707
		Among regions	5.46	0.05456	0.01857
15.CR+EDO+ON+OS vs. OYO vs. EK vs. OG	4	Within populations	95.22	0.04776	0.00000
		Within populations within regions	12.00	0.11190	0.00000
		Among regions	-7.22	-0.07222	0.99022
16.CR+EDO vs. OY+OS vs. OG vs. ON+EK	4	Within populations	91.07	0.08934	0.00000
		Within populations within regions	2.14	0.02293	1.00000
		Among regions	6.80	0.06797	0.00489
17. OY+OG vs. ON+EK+OS vs. CR vs. EDO	4	Within populations	92.49	0.07513	0.00000
		Within populations within regions	10.03	0.09784	0.00000
		Among regions	-2.52	-0.02518	0.66960
18. OY+OS+OG vs. EK+ON vs. CR vs. EDO	4	Within populations	92.23	0.07775	0.00000
		Within populations within regions	8.38	0.08327	0.99707
		Among regions	-0.60	-0.00602	0.46334
19. West African populations (overall)	1	Within populations	16.99	-	-
		Among populations	83.01	0.16988	0.00000

20. West African populations + SA (overall)	1	Within populations	38.02	-	-
		Among populations	61.98	0.61978	0.00000
21. GHANA+BENIN vs. NIG+CAM+EGUINEA	2	Within populations	83.02	0.16984	0.00000
		Within populations within regions	16.99	0.16991	0.00000
		Among regions	-0.01	-0.00008	0.30205
22. NIG vs. West African populations + SA	2	Within populations	43.71	0.56287	0.00000
		Within populations within regions	152.59	0.77732	0.00000
		Among regions	-96.31	-0.96306	1.00000
23. NIG vs. West African populations	2	Within populations	84.28	0.23894	0.00000
		Within populations within regions	26.46	0.77732	0.00000
		Among regions	-10.74	-0.10738	0.50538
24. CAM vs. West African populations + SA	2	Within populations	60.35	0.39653	0.00000
		Within populations within groups	165.28	0.73254	0.00000
		Among groups	-125.63	-1.25628	1.00000
25. CAM vs. West African populations	2	Within populations	85.41	0.14591	0.00000
		Within populations within groups	21.12	0.19822	0.00000
		Among groups	-6.53	-0.06525	0.42424
26. GHANA vs. West African populations	2	Within populations	64.62	0.35382	0.00000
		Within populations within groups	6.98	0.09755	0.00000
		Among groups	28.40	0.28398	0.10459

27. CAM+EGUINEA vs. NIG+BEN+GHANA	2	Within populations	82.95	0.17054	0.00000
		Within populations within regions	16.86	0.16893	0.00000
		Among regions	0.19	0.00195	0.30010
28. CAM+EGUINEA vs. NIG+BEN vs. GHANA	3	Within populations	78.73	0.21271	0.00000
		Within populations within regions	-0.39	-0.00501	0.28739
		Among regions	21.66	0.21664	0.03617
29. CAM+NIG+EGUINEA vs. BENIN vs. GHANA	3	Within populations	80.10	0.19899	0.00000
		Within populations within regions	12.64	0.13631	0.00000
		Among regions	7.26	0.07257	0.36559
30. NIG vs. BEN+GHANA vs. CAM+EGUINEA	3	Within populations	83.19	0.16814	0.00000
		Within populations within regions	23.77	0.22226	0.00000
		Among regions	-6.96	-0.06958	0.51320
31. GHANA vs. BENIN vs. NIG vs. CAM+EGUINEA	4	Within populations	82.86	0.17145	0.00000
		Within populations within regions	-5.43	-0.07010	0.19746
		Among regions	22.57	0.22572	0.18280

[1]Overall is Nigerian Grasscutter populations which were as follows (number of samples in parentheses): Cross River (CR; n=30), Ekiti (EK; n=12), Ogun (OG; n=10), Okomu-Edo (EDO; n=17), Ondo (ON; n=19), Osun (OS; n=11) and Oyo (OY; n=7).

[2]Remaining populations listed above except the mentioned population.

CAM – Cameroon, EGUINEA – Equatorial Guinea

Table S14: Pair-wise difference F_{ST} between subpopulations in Nigeria, Benin, Cameroon, and Ghana based on mitochondrial D-loop regions

	Benin	Cameroon	Ghana	Cross River	Ekiti	Ogun	Okomu (Edo)	Ondo	Osun	Oyo
Benin	-									
Cameroon	0.510*	-								
Ghana	0.334*	0.516*	-							
Cross River	0.292*	0.510*	0.451*	-						
Ekiti	0.045*	0.566*	0.388*	0.303	-					
Ogun	-0.020	0.535*	0.352*	0.273*	0.023	-				
Okomu (Edo)	0.197*	0.534*	0.408*	0.224*	0.326*	0.193*	-			
Ondo	0.085*	0.525*	0.390*	0.255*	0.044	-0.004	0.195*	-		
Osun	-0.029	0.566*	0.359*	0.324*	0.072*	0.031	0.289*	0.122*	-	
Oyo	0.204	0.575*	0.373*	0.410*	0.379*	0.266*	0.383*	0.281*	0.317*	-

Numbers with asterisks and highlighted in yellow background are statistically significant at $p<0.05$

Table S15: Exact test of differentiation based on haplotype frequencies for the mitochondrial D-loop region

	**Nigeria**	**Ghana**	**Cameroon**	**Cross river**	**Ondo**	**Edo**	**Benin**	**Ekiti**	**Osun**	**Ogun**	**Oyo**	**South Africa**
**Nigeria**												
**Ghana**	0.00E+00											
**Cameroon**	0.00E+00	0.00E+00										
**Cross river**	3.24E-03	0.00E+00	0.00E+00									
**Ondo**	1.56E-01	0.00E+00	0.00E+00	0.00E+00								
**Edo**	6.74E-02	0.00E+00	0.00E+00	2.40E-04	1.80E-04							
**Benin**	3.31E-02	6.35E-03	1.00E-05	0.00E+00	4.80E-04	1.00E-05						
**Ekiti**	1.90E-01	2.22E-03	0.00E+00	0.00E+00	5.25E-02	0.00E+00	4.74E-01					
**Osun**	2.36E-01	2.07E-02	3.00E-05	0.00E+00	7.23E-03	2.30E-04	3.31E-01	4.16E-01				
**Ogun**	4.92E-01	1.46E-02	5.00E-05	0.00E+00	2.36E-01	5.00E-05	3.42E-01	7.42E-01	4.05E-01			
**Oyo**	1.53E-01	2.29E-02	7.70E-04	0.00E+00	1.21E-02	5.00E-05	9.71E-02	1.11E-01	5.70E-02	1.28E-01		
**South Africa**	1.09E-02	3.83E-02	4.06E-02	2.10E-03	9.12E-02	3.02E-02	7.88E-02	9.87E-02	3.02E-02	7.63E-02	2.79E-02	

The test was conducted in Arlequin using 1,000,000 steps of Markov chain (Raymond and Rousset 1995)

Table S16: Pair wise F_{ST} values of NIG+CAM+BEN+EGUINEA+SA-Grasscutter populations-(*CYTB*)

**Population**	**Nigeria**	**South Africa**	**Equatorial Guinea**	**Cameroon**	**Benin**	**Ghana**
Nigeria	-					
South Africa	0.942*	-				
Equatorial Guinea	0.209*	1.000	-			
Cameroon	0.111*	0.986*	0.258	-		
Benin	-0.003	0.993*	0.790*	0.304*	-	
Ghana	0.291*	0.982*	0.709*	0.641*	0.599*	-

F_{ST} P values significance level 0.05.

Table S17: *CYTB* haplotype sharing among grasscutter populations from Nigeria, Ghana, Benin, Cameroon, Equatorial Guinea, and South Africa

HT	HT Name	Frequency	Nigeria	Ghana	Benin	Cameroon	Equatorial Guinea	South Africa
CYHT1	**53-OY**	**103**	73	0	14	16	0	0
CYHT2	**72-OG**	**1**	1	0	0	0	0	0
CYHT3	**74-OG**	**1**	1	0	0	0	0	0
CYHT4	**69-OG**	**23**	23	0	0	0	0	0
CYHT5	**77-EK**	**1**	1	0	0	0	0	0
CYHT6	**50-OK**	**1**	1	0	0	0	0	0
CYHT7	**104-ON**	**4**	4	0	0	0	0	0
CYHT8	**131-CAM**	**15**	0	0	0	13	2	0
CYHT9	**KJ742647-SA**	**3**	0	0	0	0	0	3
CYHT11	**KJ193488T1332-GHA**	**7**	0	7	0	0	0	0
CYHT12	**KJ193487T1323-GHA**	**3**	0	2	1	0	0	0
CYHT13	**KJ193479T1297-GHA**	**1**	0	1	0	0	0	0
CYHT14	**55-OY**	**1**	1	0	0	0	0	0
